# A Synthetic Plasmid Toolkit for *Shewanella oneidensis* MR-1

**DOI:** 10.3389/fmicb.2019.00410

**Published:** 2019-03-08

**Authors:** Yingxiu Cao, Mengyuan Song, Feng Li, Congfa Li, Xue Lin, Yaru Chen, Yuanyuan Chen, Jing Xu, Qian Ding, Hao Song

**Affiliations:** ^1^Key Laboratory of Systems Bioengineering (Ministry of Education), SynBio Research Platform, Collaborative Innovation Center of Chemical Science and Engineering (Tianjin), School of Chemical Engineering and Technology, Tianjin University, Tianjin, China; ^2^College of Food Science and Technology, Hainan University, Haikou, China

**Keywords:** *Shewanella oneidensis* MR-1, plasmid toolkit, BioBrick, *c*-type cytochrome, fine-tuning, synthetic biology

## Abstract

*Shewanella oneidensis* MR-1 is a platform microorganism for understanding extracellular electron transfer (EET) with a fully sequenced and annotated genome. In comparison to other model microorganisms such as *Escherichia coli*, the available plasmid parts (such as promoters and replicons) are not sufficient to conveniently and quickly fine-tune the expression of multiple genes in *S. oneidensis* MR-1. Here, we constructed and characterized a plasmid toolkit that contains a set of expression vectors with a combination of promoters, replicons, antibiotic resistance genes, and an RK2 origin of transfer (*oriT*) cassette, in which each element can be easily changed by fixed restriction enzyme sites. The expression cassette is also compatible with BioBrick synthetic biology standards. Using green fluorescent protein (GFP) as a reporter, we tested and quantified the strength of promoters. The copy number of different replicons was also measured by real-time quantitative PCR. We further transformed two compatible plasmids with different antibiotic resistance genes into the recombinant *S. oneidensis* MR-1, enabling control over the expression of two different fluorescent proteins. This plasmid toolkit was further used for overexpression of the MtrCAB porin-*c*-type cytochrome complex in the *S. oneidensis* Δ*mtrA* strain. Tungsten trioxide (WO_3_) reduction and microbial fuel cell (MFC) assays revealed that the EET efficiency was improved most significantly when MtrCAB was expressed at a moderate level, thus demonstrating the utility of the plasmid toolkit in the EET regulation in *S. oneidensis*. The plasmid toolkit developed in this study is useful for rapid and convenient fine-tuning of gene expression and enhances the ability to genetically manipulate *S. oneidensis* MR-1.

## Introduction

*Shewanella oneidensis* MR-1, a Gram-negative exoelectrogen, is regarded as an important model microorganism with extracellular electron transfer (EET) pathways for bio-electrochemical applications in environmental and energy fields, including bioelectricity generation and production of chemicals (Hou et al., 2009, 2011; Kouzuma et al., 2015; Shi et al., 2016; Kumar et al., 2017; Li et al., 2018b). After fully sequencing and annotating its genome (Venkateswaran et al., 1999; Heidelberg et al., 2002; Daraselia et al., 2003), a number of efforts have been made to engineer EET in *S. oneidensis*. For example, disruption of the putative cell surface polysaccharide biosynthesis gene *SO3177* (Kouzuma et al., 2010) or overexpression of the *c*-type cytochrome gene *mtrC* (Bretschger et al., 2008) were undertaken to generate higher current output. With the advancement of synthetic biology, multiple genes were simultaneously manipulated to facilitate EET in *Shewanella*. Golitsch et al. (2013) used an arabinose-inducible promoter pBAD to upregulate the expression of an operon composed of the genes *mtrF*, *mtrA*, and *mtrB*, which had previously been deleted from the genome in *S. oneidensis*, showing that modulation of the operon expression levels is an efficient strategy in enhancing ferric iron reduction capacity and anode reduction performance. West et al. (2017) characterized a native inducible expression system induced by trimethylamine N-oxide (TMAO) in *S. oneidensis* to control EET, which was then successfully used to regulate EET by inducing *mtrCAB* gene expression with TMAO. The ability to induce this pathway was assessed by measuring iron reduction over time and by analyzing anodic current produced by cells grown in bioreactors. Yang et al. (2015) heterologously expressed a synthetic flavin biosynthesis pathway from *Bacillus subtilis* in the BioBrick compatible vector pYYDT, derived from the plasmid pHG101, to enhance the rate of EET in engineered *S. oneidensis* MR-1. By using this plasmid, Li et al. (2018a) assembled four genes that are mostly responsible for increasing NADH regeneration in *S. oneidensis* MR-1.

Improved genetic engineering strategies could provide additional opportunities to modulate the metabolism of microorganisms (Lin et al., 2018). Although manipulation of multiple genes has been employed to engineer *S. oneidensis*, the availability of gene expression tools is still not as abundant as other well-studied platform microorganisms, such as *Escherichia coli* or *Saccharomyces cerevisiae* (Keasling, 2012; Chae et al., 2017). In *E. coli*, a large number of expression and regulatory elements make the fine-tuning of gene expression quick and convenient. Researchers have combined various biological parts in *E. coli*, such as promoters with different strengths (e.g., pBAD, pTrc, pT5, pT7, placUV5, pLtetO-1, and double tac), replication origins (e.g., p15A, ColE, pBBR1, pSC101, and pBR322), antibiotic resistance markers [e.g., chloramphenicol (CmR), kanamycin (Kan), carbenicillin (Cb), ampicillin (Amp), and spectinomycin (Spect)], to fine-tune gene expression, which led to significant increases in the production of taxadiene (Ajikumar et al., 2010), *n*-butanol (Bond-Watts et al., 2011), L-tyrosine (Juminaga et al., 2012), and amorphadiene (Anthony et al., 2009). Heterologous expression of MtrCAB under the control of the T7 promoter in *E. coli* could create an artificial electrogenic cell (Jensen et al., 2010), but high MtrCAB expression in the presence of the Isopropyl β-D-Thiogalactoside (IPTG) inducer may impair EET efficiency in *E. coli*. Goldbeck et al. (2013) used *E. coli* with a more tunable induction system and a panel of constitutive promoters to express the MtrCAB pathway. They found that the efficiency of MtrC and MtrA synthesis decreased when controlled by strong promoters, leading to a significant decrease in EET efficiency. To improve the ability of manipulating *S. oneidensis* MR-1, more vectors were developed. Hajimorad and Gralnick (2016) demonstrated that different replication origins (e.g., p15A, pMB1, and pBBR1), selection markers (e.g., Kan and CmR), and an RK2 *oriT* cassette could be used in *S. oneidensis* MR-1, which provided a useful tool in engineering *S. oneidensis*. Continued development of synthetic biology parts, coupled with quantitative characterization, will enable more delicate or complicated engineering of *S. oneidensis*.

**FIGURE 1:**
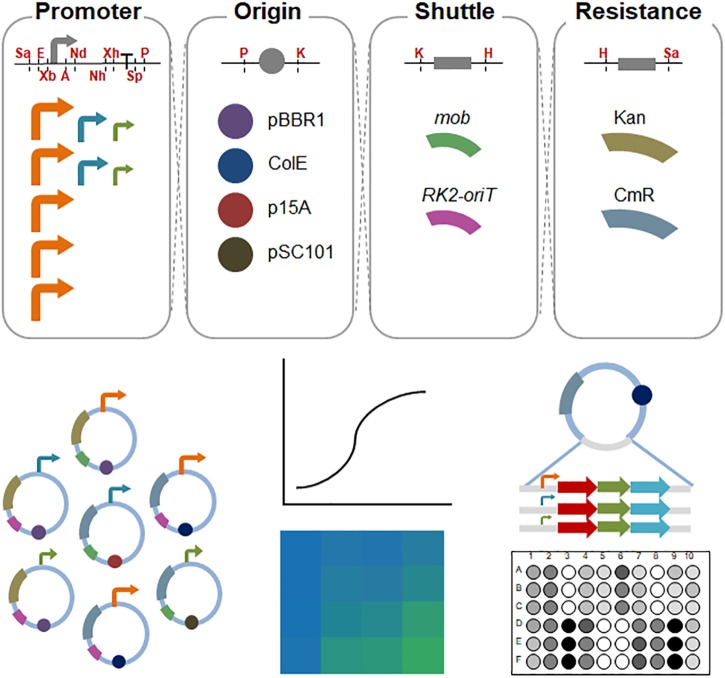
Schematic diagram of the assembled shuttle vectors in *S. oneidensis* MR-1. The cutting sites of the restriction enzymes are shown in red. Sa, *Sac*I; E, *Eco*RI; Xb, *Xba*I; A, *Avr*II; Nd, *Nde*I; Nh, *Nhe*I; Xh, *Xho*I; Sp, *Spe*I; P, *Pst*I; K, *Kpn*I; H, *Hind*III. High-strength promoters (i.e., pBAD, pXyl, pCI, pJ23119, and pTac) are shown in orange; medium-strength promoters (pTet and pTrc^∗^) are shown in aqua; and low-strength promoters (placUV5 and pLlacO1) are shown in olive.

Here, we report the development of a synthetic plasmid toolkit for *S. oneidensis* MR-1. A number of available biological parts, such as promoters, replication origins, a conjugal transfer shuttle cassette, and antibiotic resistance genes were tested to design and construct easily available and compatible vectors for *Shewanella* (Figure 1). Firstly, we evaluated and characterized the strength of various promoters in *E. coli* and *S. oneidensis* MR-1 using the green fluorescent protein (GFP) as a reporter. Furthermore, we characterized the strength of inducible promoters by regulating inducer concentrations and induction times. Secondly, we quantified the copy numbers of different replicons in *S. oneidensis* MR-1 by real-time quantitative PCR (RT-qPCR) and found that copy number was directly correlated with the fluorescence intensity of GFP. Additionally, we simultaneously transformed two compatible plasmids into recombinant *S. oneidensis* MR-1, thus achieving fine control of the expression of two different fluorescent proteins [GFP and blue fluorescent protein (BFP)/superfolder cyan fluorescent protein (CFP)] through modulation of inducer concentration or the use of promoters with different strengths. Lastly, we took the porin-cytochrome complex MtrCAB for an example to demonstrate that EET efficiency can be fine-tuned in recombinant *S. oneidensis*Δ*mtrA* strain by the plasmid toolkit we developed in this study. The plasmid toolkit developed in this study enabled rapid and convenient fine-tuning of gene expression with greater controllability and predictability, which will accelerate future genetic manipulations of *S. oneidensis* MR-1.

## Materials and Methods

### Strains and Growth Conditions

All bacterial strains used in this study are listed in Table 1. For genetic manipulation, *E. coli* Trans1-T1, BL21, MG1655, and WM3064 were cultivated in Luria-Bertani (LB) medium at 37°C. The wild-type and recombinant *S. oneidensis* MR-1 strains were cultured in LB medium at 30°C. Unless otherwise specified, genetic manipulation antibiotics were used at the following concentrations: kanamycin at 50 μg/ml and chloramphenicol at 34 μg/ml. The *E. coli* WM3064 culture was supplemented with 0.3 mM 2,6-Diaminopimelic acid (DAP).

**Table 1:** Strains and plasmids used in the study.

Strain	Description	Source
*E. coli* MG1655	Wild type	Lab stock
*S. oneidensis* MR-1	Wild type	Lab stock
*E. coli* WM3064	A DPA auxotroph of *E. coli* could transfer plasmid into *S. oneidensis* MR-1 by conjugation	Lab stock
*E. coli* Trans1 T1	Cloning strain	TransGen
*E. coli* BL21(DE3)	Cloning strain	TransGen
*S-*Δ*mtrA*	A *mtrA* deletion mutant of *S. oneidensis* MR-1	Lab stock
L-*mtrCAB*	*S-*Δ*mtrA* carrying pHG13-placUV5-CoIE-MtrCAB	This study
M-*mtrCAB*	*S*-Δ*mtrA* carrying pHG13-pTet-CoIE-MtrCAB	This study
H-*mtrCAB*	*S-*Δ*mtrA* carrying pHG13-pBAD-CoIE-MtrCAB	This study

**Plasmid**	**Description**	**Source**

pHG11	*rep*^pBBR1^, KanR without PstI restriction site, Mob without BsaI restriction site	Lab stock
pHG12	*rep*^pBBR1^, KanR without PstI and NcoI restriction sites, Mob without BsaI restriction site, with insertion of SacI in front of EcoRI restriction site.	This study
pHG12-pBAD	pBAD, *rep*^pBBR1^, KanR, Mob	This study
pHG12-pXyl	pXyl, *rep*^pBBR1^, KanR, Mob	This study
pHG12-pTet	pTet, *rep*^pBBR1^, KanR, Mob	This study
pHG12-pTrc^∗^	pTrc^∗^, *rep*^pBBR1^, KanR, Mob	This study
pHG12-pTac	pTac, *rep*^pBBR1^, KanR, Mob	This study
pHG12-pLlacO1	pLlacO1, *rep*^pBBR1^, KanR, Mob	This study
pHG12-placUV5	placUV5, *rep*^pBBR1^, KanR, Mob	This study
pHG12-pCI	pCI, *rep*^pBBR1^, KanR, Mob	This study
pHG12-pJ23119	pJ23119, *rep*^pBBR1^, KanR, Mob	This study
pHG12-pBAD-GFP	pBAD, *rep*^pBBR1^, KanR, Mob, *gfp* amplified from pLac-GFP	This study
pHG12-pXyl-GFP	pXyl, *rep*^pBBR1^, KanR, Mob, *gfp*	This study
pHG12-pTet-GFP	pTet, *rep*^pBBR1^, KanR, Mob, *gfp*	This study
pHG12-pTrc^∗^-GFP	pTrc^∗^, *rep*^pBBR1^, KanR, Mob, *gfp*	This study
pHG12-pTac-GFP	pTac, *rep*^pBBR1^, KanR, Mob, *gfp*	This study
pHG12-pLlacO1-GFP	pLlacO1, *rep*^pBBR1^, KanR, Mob, *gfp*	This study
pHG12-placUV5-GFP	placUV5, *rep*^pBBR1^, KanR, Mob, *gfp*	This study
pHG12-pCI-GFP	pCI, *rep*^pBBR1^, KanR, Mob, *gfp*	This study
pHG12-pJ23119-GFP	pJ23119, *rep*^pBBR1^, KanR, Mob, *gfp*	This study
pHG13-pTrc^∗^-CoIE-GFP	pTrc^∗^, *rep*^ColE^, CmR, *gfp*, *oriT*	This study
pHG13-pTrc^∗^-p15A-GFP	pTrc^∗^, *rep*^p15A^, CmR, *gfp*, *oriT*	This study
pHG13-pTrc^∗^-pSC101-GFP	pTrc^∗^, *rep*^pSC101^, CmR, *gfp*, *oriT*	This study
pHG13-pTrc^∗^-CoIE-BFP	pTrc^∗^, *rep*^ColE^, CmR, *oriT*, BFP	This study
pHG13-pTrc^∗^-CoIE-CFP	pTrc^∗^, *rep*^ColE^, CmR, *oriT*, CFP	This study
pHG13-pBAD-CoIE-BFP	pBAD, *rep*^ColE^, CmR, *oriT*, BFP	This study
pHG13-pBAD-CoIE-CFP	pBAD *rep*^ColE^, CmR, *oriT*, CFP	This study
pHG13-pTet-CoIE-BFP	pTet, *rep*^ColE^, CmR, *oriT*, BFP	This study
pHG13-placUV5-CoIE-BFP	placUV5, *rep*^ColE^, CmR, *oriT*, BFP	This study
pHG13-pBAD-CoIE- MtrCAB	pBAD, *rep*^ColE^, CmR, *oriT*, mtrA, mtrC, mtrB	This study
pHG13-pTet-CoIE-MtrCAB	pTet,*rep*^ColE^, CmR, *oriT*, mtrA, mtrC, mtrB	This study
pHG13-placUV5-CoIE- MtrCAB	placUV5, *rep*^ColE^, CmR, *oriT*, mtrA, mtrC, mtrB	This study

### Plasmid Constructions

All the plasmids used in this study are listed in Table 1. Plasmid construction processes were performed in *E. coli* Trans1-T1 and *E. coli* BL21. The plasmid is composed of four elements: replication origin, antibiotic resistance gene, conjugal transfer shuttle cassette, and expression cassette, with fixed restriction enzyme sites between each part as shown in Figure 1 and Supplementary Figure S1. The sequences of each biological element and the corresponding restriction enzyme sites were synthesized by Genewiz (China), which are listed in Supplementary Table S2. Specifically, plasmid pHG12 was constructed from plasmid pHG11 (Cao et al., 2017) by site-directed mutagenesis of the NcoI restriction site and insertion of the SacI in front of EcoRI restriction site. The primers used are presented in Supplementary Table S1.

The sequences of green, blue and superfolder cyan fluorescent protein gens (*gfp*, *bfp*, and *cfp*) were codon-optimized and synthesized by Genewiz (China), which were listed in Supplementary Table S2. They were digested with NdeI/XhoI and inserted into the corresponding sites of the expression cassette in relative plasmid. The MtrCAB clusters were amplified from the *S. oneidensis* MR-1 genome using MtrCAB-F/MtrCAB-R primer pairs, listed in Supplementary Table S1, which were then digested with NdeI/XhoI and inserted into the corresponding sites of the plasmids carrying the promoter pBAD, pTet, and placUV5, respectively, resulting in three *mtrCAB* expression vectors.

### Conjugation Assay

Transformed into *S. oneidensis* MR-1 or *S. oneidensis*Δ*mtrA* strains (Cao et al., 2017), plasmids were firstly transformed into the plasmid donor cell *E. coli* WM3064 (a DAP auxotroph) and then transferred into *S. oneidensis* MR-1 or *S. oneidensis* Δ*mtrA* by conjugation. For the growth of *E. coli* WM3064 that has RP4 Tra function integrated into the chromosome, 0.3 mM 2,6-Diaminopimelic acid (DAP) was needed in the culture medium. In brief, 500 μl donor cells and 500 μl recipient cells were mixed and then the mixture was collected by centrifugation (5000 rpm for 10 min). Cells were suspended with 1 ml LB medium containing DAP and incubated for 1 h at 30°C. The appropriate volume of cells was next spread on LB plates with appropriate antibiotics and placed overnight in 30°C incubator to allow conjugation. To arrive at multiple-plasmid *Shewanella* strains, *S. oneidensis* MR-1 cells harboring a single-plasmid served as the recipient cell in the above steps.

### Fluorescence Assay

Green, blue, and superfolder cyan fluorescent proteins (GFP, BFP, and CFP) were used to characterize the expression intensity. A total of 0.1 mM IPTG, 10 mM arabinose, 1 g/l xylose, or 100 ng/ml anhydrotetracycline hydrochloride (aTc) was used as the normal concentration for inducing the corresponding promoters. For concentration-response and time-response assays, GFP intensities in *S. oneidensis* MR-1 were tested with a series of inducer concentrations. IPTG, arabinose, and xylose were diluted to 10^-3^–10^4^ μM, 10^-2^–10^5^ μM, and 10^-5^–10^1^ g/l using 10-fold serial dilutions, respectively. Additionally, aTc was used at the following concentrations (ng/ml): 25, 50, 100, 250, 500, 1000, 1500, 2000, and 2500. To simultaneously fine-tune the expression of multiple fluorescent proteins in *S. oneidensis* MR-1, we adjusted inducer concentrations for IPTG-inducible pTac or arabinose-inducible pBAD promoters. IPTG was used at the following concentrations (μM): 1, 12.5, 15, and 100. Arabinose was used at the following concentrations (mM): 0.1, 0.25, 0.5, and 1.

For fluorescence intensity assays, each strain was inoculated from a freshly transformed single colony on an LB agar plate into 2 ml LB medium as a seed culture. When cell accumulation reached stationary phase (assessed spectrophotometrically at OD_600_), 50 μl of seed culture was re-inoculated in test tubes containing 5 ml fresh LB medium supplemented with the appropriate antibiotic and the corresponding concentration of inducer. After 24 h of growth at 30°C with constant shaking (200 rpm), 200 μl suspensions from each test tube were centrifuged at 4000 rpm for 2 min to remove the supernatant, after which the culture was washed and resuspended in phosphate-buffered saline (PBS) and transferred to a 96-well polystyrene plate (black plate, clear bottom) (Corning Incorporated 3603, United States). Cell optical density and fluorescence intensity were detected by a SpectraMax M2 microplate reader. Optical density was measured at 600 nm. The excitation/emission wavelengths for GFP, BFP, and CFP were set at 485 nm/520 nm, 399 nm/456 nm, and 425 nm/475 nm, respectively. Assays were performed in triplicate, and untransformed *E. coli* MG1655 or *S. oneidensis* MR-1 strains were used as controls. Relative fluorescence was calculated as the fluorescence per OD_600_ for each strain subtracted from that of the untransformed *E. coli* MG1655 or untransformed *S. oneidensis* MR-1 control:

$$\begin{array}{c} \text{Relative Fluorescence}\\ =\frac{\text{Fluorescence }(\text{i})}{\text{OD }(\text{i})}-\frac{\text{Fluorescence }(\text{control})}{\text{OD }(\text{control})} \end{array}$$

### Determination of Replicon Copy Number by Real-Time Quantitative PCR (RT-qPCR)

#### Preparation of Template DNA for qPCR

Recombinant *S. oneidensis* MR-1 isolates harboring different plasmids (e.g., pHG13-pTrc^∗^-CoIE-GFP, pHG13-pTrc^∗^-p15A-GFP, pHG13-pTrc^∗^-pSC101-GFP, and pHG12-pTrc^∗^-GFP) were cultivated to exponential growth phase, after which total DNA was extracted using the Bacterial Genome DNA extraction kit (Kang Wei Century, China) according to the manufacturer’s instructions. Template DNA extracted from the recombinant *S. oneidensis* MR-1 was normalized to 2 ng/μl for consistency in the RT-qPCR assay (Lee et al., 2006).

#### Construction of Standards for RT-qPCR

The sequences of the four replicons (CoIE, p15A, pSC101, and pBBR1) were amplified using primers listed in Supplementary Table S1. The products were separately cloned into T vectors using a Puc-T TA cloning kit (Kang Wei Century, China). Each cloned plasmid was purified using the TIANquick Mini Purification kit (Tiangen, Beijing, China) and was used as a standard (Dhanasekaran et al., 2010). RT-qPCR was carried out using a real-time fluorescent quantitative PCR machine (Applied Biosystems) in white-walled PCR plates (96 wells). SYBR^®^ Premix Ex Taq^TM^ II (Tli RNaseH Plus) and ROXplus (TaKaRa) were used to make a Master Mix, which also contained TaKaRa Ex Taq HS, a dNTP mixture, Mg^2+^, Tli RNaseH, TB Green, and ROX Reference Dye. Reactions were prepared in a total volume of 18 μl, containing 10 μM of each standard RT-qPCR primer, 2× Master Mix, sterile water and 2 μl cDNA. The cycle conditions were set as follows: initial template denaturation at 95°C for 30 s, followed by 40 cycles of denaturation at 95°C for 5 s, and combined primer annealing/elongation at 60°C for 40 s, with the fluorescence data acquired at 60°C and analyzed using 7500 Software V2.0.4 (Applied Biosystems).

#### Construction of Standard Curves and Absolute Quantification

A 10-fold serial dilution series of each standard plasmid, ranging from 1 × 10^3^ to 1 × 10^8^ copies/μl, was used to construct the standard curves. The concentration of the plasmid was measured using a spectrophotometer (Thermo Scientific), and the corresponding copy number was calculated using the following equation (Whelan et al., 2003):

$$\text{DNA }(\text{copy})=\frac{6.02\times 10^{23}(\text{copy/mol})\times \text{DNA amount }(\text{g})}{\text{DNA length (dp)}\times \text{660 (g/mol/dp)}}$$

The threshold cycle (*C*_T_) values for each dilution were measured in triplicate by RT-qPCR with the CoIE-, p15A-, pSC101-, and pBBR1-sets to generate the corresponding standard curves. Standard curves were obtained by plotting the threshold cycle (*C*_T_) on the y-axis and the natural log of concentration (copies/μl) on the *x*-axis. *C*_T_, slope, PCR efficiency, correlation coefficient (*R*^2^), and percentage of variance in copy numbers were calculated using the default settings of 7500 Software V2.0.4 (Applied Biosystems). Absolute quantification of the copy numbers of target replicon genes were obtained by comparing the *C*_T_ value of each template DNA sample to a standard curve (Yu et al., 2005).

### Tungsten Trioxide Reduction Assay

This assay was performed according to Yuan et al. (2014). The process for the tungsten trioxide (WO_3_) nanorod assembly synthesis solution is as follows: Dilute 0.825 g of Na_2_WO_4_ ⋅ 2H_2_O and 0.290 g of NaCl in 20 ml of sterile water. Add 3 M HCl slowly under stirring until the pH reaches 2.0. This solution needs to be freshly prepared. The process for the sodium lactate minimal salt medium is as follows: Per liter, dissolve 2.02 g of sodium lactate, 5.85 g of NaCl, 11.91 g of HEPES, 0.3 g of NaOH, 1.498 g of NH_4_Cl, 0.097 g of KCl, 0.67 g of NaH_2_PO_4_ ⋅ 2H_2_O and 1 ml per liter trace mineral stack solution (containing per liter: 1.5 g of C_6_H_9_NO_6_, 30 g of MgSO_4_ ⋅ 7H_2_O, 5 g of MnSO_4_ ⋅ H_2_O, 10 g of NaCl, 1 g of FeSO_4_ ⋅ 7H_2_O, 1 g of CaCl_2_ ⋅ 2H_2_O, 1 g of CoCl_2_ ⋅ 6H_2_O, 1.3 g of ZnCl_2_, 0.1 g of CuSO_4_ ⋅ 5H_2_O, 0.1 g of AlK(SO_4_)_2_ ⋅ 12H_2_O, 0.1 g of H_3_BO_3_, 0.25 g of Na_2_MoO_4_ ⋅ 2H_2_O, 0.25 g of NiCl_2_ ⋅ 6H_2_O, and 0.25 g of Na_2_WO_4_ ⋅ 2H_2_O) in sterile water and autoclave the mixture immediately. For the WO_3_ nanorod assembly containing sodium lactate minimal salt suspension, the process is as follows: dissolve 5 g per liter of WO_3_ nanorod assembly in sodium lactate minimal salt suspension and autoclave the resulting suspension immediately.

Overexpression of the *c*-type cytochromes, MtrCAB, in engineered *S. oneidensis* Δ*mtrA* was tested under the control of placUV5, pTet, and pBAD promoters. The parental and recombinant strains were named as *S-ΔmtrA*, L-*mtrCAB*, M-*mtrCAB*, and H-*mtrCAB*, respectively, according to the expression level of the gene (i.e., L, low; M, medium; H, high). They were activated overnight in LB medium with or without chloramphenicol. Then, 50 μl from each bacterial culture suspension from a 2 ml LB medium seed culture (originating from a freshly transformed single colony on a LB agar plate) was used to inoculate a test tube containing 5 ml fresh LB medium supplemented with or without chloramphenicol. The inducers for each promoter (i.e., 0.1 mM IPTG for placUV5, 1,000 ng/ml aTc for pTet, and 10 mM arabinose for pBAD) were added to express *mtrCAB* when OD_600_ reached ∼0.5. After 24 h of induction, cells were collected by centrifugation at 4000 rpm at 4°C for 5 min, after which the pellets were resuspended in sterile sodium lactate minimal salt medium, with appropriate dilution to achieve an OD_600_ ∼0.8. Then, 100 μl of the above resuspended bacterial solution and 80 μl of 5 g/l sterile WO_3_ nanorod assembly containing sodium lactate minimal salt suspension were mixed completely before being added into a 96-well plate. After that, 80 μl of petrolatum oil was added into each well immediately to ensure anaerobic conditions for the bioelectrochromic reaction, and the plate was incubated at 30°C in an incubator. We observed conspicuous color development in the plate after 30 min, but maintained incubation for 6 h, after which we transferred the plate into a scanner. As the plate was being scanned, an opaque box was used to shield it from external light. Then, the average color intensity of each well was determined by analyzing their ‘Density (mean)’ of the area of interest in the scanned photograph with the ImageJ Software V.1.8.0. Culture medium without bacteria was used as a control to ensure that the WO_3_ electrochromic reaction was not caused by the culture medium. Triplicate experiments were performed for each strain.

### MFC Setup and Electrochemical Analysis

Overnight *S-*Δ*mtrA*, L-*mtrCAB*, M-*mtrCAB*, and H-*mtrCAB* culture suspensions were inoculated into 100 ml fresh LB broth at 30°C with shaking (200 rpm). After around 10 h culture, the amplification-cultured cells were subsequently centrifuged and collected at 4°C and underwent 6000 rpm for 10 min to remove the supernatant. The concentrations of cell suspensions were adjusted to the same level (OD_600_ ≈ 0.5), and the suspensions were dispersed into the anode chambers. In this MFC, *S-*Δ*mtrA* was used as a control. Whenever needed, 34 μg/ml chloramphenicol was added in anolytes to ensure consistent culture condition. For recombinant *S. oneidensis*Δ*mtrA* strain harboring the plasmid under the control of the inducible promoter, the medium was supplemented with 0.1 mM IPTG for promoter placUV5, 1,000 ng/ml aTc for pTet, or 10 mM arabinose for pBAD. All MFCs were incubated in a 30°C incubator and each group was tripled for parallel experiments. Dual-chamber MFCs with a working volume of 110 mL separated by the Nafion 117 membrane (DuPont Inc., United States) were applied in this study. Carbon cloth was used as the electrodes for both anode (2.5 cm × 2.5 cm, i.e., the geometric area is 6.25 cm^2^) and cathode (2.5 cm × 3 cm). The anolyte consisted of M9 buffer (Na_2_HPO_4_, 6 g/l; KH_2_PO_4_, 3 g/l; NaCl, 0.5 g/l; NH_4_Cl, 1 g/l; MgSO_4_, 1 mM; CaCl_2_, 0.1 mM), supplemented with 18 mM lactate and 5% (v/v) LB broth. The catholyte was made of 50 mM K_3_[Fe (CN)_6_] in 50 mM KH_2_PO_4_ and 50 mM K_2_HPO_4_ solution. To measure the voltage generation, a 2 kΩ external resistor was connected into the external circuit of MFCs, and the output voltages were recorded across the external loading resistor with a digital multimeter (DT9205A). Linear sweep voltammetry (LSV) analysis with a scan rate (0.1 mV/s) was conducted on a two-electrode mode, where the anode performed as the working electrode and the cathode as the reference as well as the counter electrode to obtain the polarization curves to estimate the maximum power density (the potential decreased from the open circuit potential (OCP) to around -0.3 V). Power density (P) was calculated as P = V (output voltage) × I (current density). Both I and P were normalized to the projected area of the anode surface.

## Results and Discussion

### Characterization of Promoters in *S. oneidensis* MR-1

**FIGURE 2:**
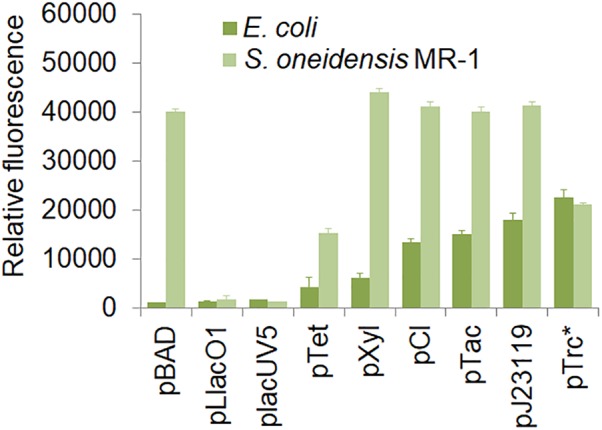
Characterization of various promoters in *S. oneidensis* MR-1. *E. coli* MG155 and *S. oneidensis* MR-1 harbored a single plasmid containing the pBAD, pLlacO1, placUV5, pTet, pXyl, pCI, pTac, pJ23119, and pTrc^∗^(no repressor lacO) promoters, respectively. The error bars (mean ± SD) were derived from triplicate experiments for each strain.

In this study, we designed a number of shuttle vectors, in which each element could be constructed or replaced using fixed restriction enzyme sites (Figure 1 and Supplementary Figure S1). Firstly, we characterized 9 promoters in *S. oneidensis* MR-1, which included pBAD, pLlacO1, placUV5, pTet, pXyl, pCI, pTac, pJ23119, and pTrc^∗^. These promoters could be classified into two categories: constitutive promoters (e.g., pCI, pJ23119, and pTrc^∗^) and inducible promoters (e.g., IPTG-inducible pTac, IPTG-inducible pLlacO1, IPTG-inducible placUV5, arabinose-inducible pBAD, xylose-inducible pXyl, and aTc-inducible pTet). The strength of these promoters was characterized using GFP as a reporter in *E. coli* MG1655 and *S. oneidensis* MR-1. 0.1 mM IPTG, 10 mM arabinose, 1 g/l xylose, or 100 ng/ml aTc was used to induce the corresponding promoters (Figure 2). In *E. coli* MG1655, the relative fluorescence showed a ladder-like distribution, which ranged from about 1,000 to more than 20,000 caused by the different expression strengths of the promoters (from low to high: pBAD, pLlacO1, placUV5, pTet, pXyl, pCI, pTac, pJ23119, and pTrc^∗^). However, the relative fluorescence intensities revealed only three expression levels in *S. oneidensis* MR-1. The pLlacO1 and placUV5 promoters showed low expression levels (1,000 ∼ 2,000); pTet and pTrc^∗^ showed medium expression levels (∼20,000); pBAD, pXyl, pCI, pJ23119, and pTac showed substantially higher expression levels (∼40,000). The highest fluorescence level was 40-fold higher than the lowest fluorescence level for *S. oneidensis* MR-1. Furthermore, the highest fluorescence intensity observed in *S. oneidensis* MR-1 was ∼2-fold higher than that in *E. coli*.

**FIGURE 3:**
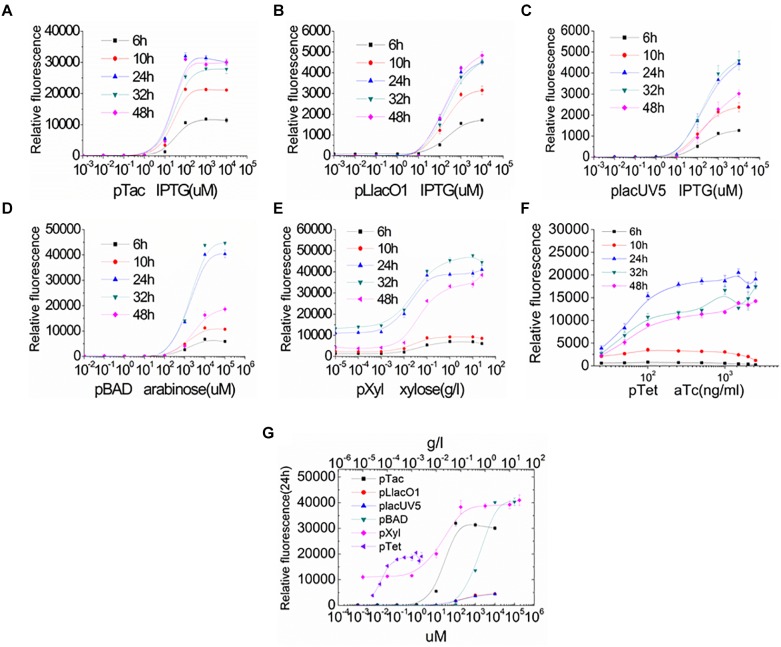
Characterization of the inducible promoters by regulating inducer concentration and induction time in *S. oneidensis* MR-1. **(A–F)** GFP expression under the control of inducible promoters was measured at 6, 10, 24, 32, and 48 h after induction with a series of different inducer concentrations. **(A–C)** IPTG-inducible pTac, pLlacO1, and placUV5; **(D)** arabinose-inducible pBAD; **(E)** xylose-inducible pXyl; **(F)** aTc-inducible pTet). **(G)** GFP expression was measured at 24 h after induction with different inducer concentrations. The error bars (mean ± SD) were derived from triplicate experiments for each strain.

To further assess the levels of gene expression in *S. oneidensis* MR-1, the inducible promoters were characterized by adjusting inducer concentration and induction time (Figure 3). The distribution of relative fluorescence intensities was narrow in the early stage of induction but became broader with increasing inducer concentrations and longer induction times. With increasing inducer concentrations, GFP expression increased until a plateau level was reached for each promoter. All promoters resulted in a characteristic sigmoidal “S”-shaped curve. For all inducible promoters, the higher expression levels were achieved at the normal inducer concentrations. Almost full induction of the pTac, pLlacO1, and placUV5 promoters occurred at IPTG concentrations of ∼0.1, ∼1, and ∼1 mM, respectively (Figure 3A–C). pBAD and pXyl were fully induced at an arabinose or a xylose concentration of about 10 mM or 1 g/l, respectively (Figure 3D,E). The pTet promoter resulted in a higher expression at an aTc concentration of about 100 ng/ml (Figure 3F). The IPTG-inducible pTac, pLlacO1, and placUV5 promoters and the arabinose-inducible pBAD promoter could tightly control gene expression, since GFP fluorescence was basically not observed at low inducer concentrations. This feature is particularly important for the expression of genes whose products may be toxic, such that expression can occur at very low levels. In contrast, we found that the xylose-inducible pXyl and the aTc-inducible pTet promoters were susceptible to leaky expression. In addition, the inducible promoters were not only regulated by inducer concentration, but were also influenced by induction time. At the beginning, the fluorescence intensities were enhanced with longer induction times, but GFP fluorescence eventually reached a higher and more stable level by 24 h. The relative fluorescence intensities of all promoters at various inducer concentrations were compared after 24 h of incubation (Figure 3G), allowing for easy selection of promoters and inducer concentrations depending on the experimental needs.

It is worth mentioning that for expression cassettes, we designed and constructed biological parts that conformed to BioBrick standards (Canton et al., 2008; Shetty et al., 2008). BioBrick vectors feature four isocaudomer pairs (*Avr*II, *Nde*I, *Xba*I, and *Spe*I), can form the monocistron or polycistron, and support the modular assembly of numbers of molecular components and multigene pathways, collectively contributing to convenient and rapid gene combination and plasmid construction (Supplementary Figure S2).

### Characterization of Antibiotic Resistance Genes, Replication Origins, and Coexistence of Two Plasmids in *S. oneidensis*

For fine-tuning the expression of multiple genes, genes should be expressed from two separate plasmids. As such, knowledge of compatible replication origins, different antibiotic resistances, and the shuttle component involved in conjugation and transformation in *S. oneidensis* MR-1 are required.

We first assessed the ability of *S. oneidensis* MR-1 to resist different antibiotics to identify suitable resistance markers for maintaining multiple plasmids. Sensitivities of the wild-type *S. oneidensis* MR-1 to three antibiotics (kanamycin at 50 or 100 μg/ml, chloramphenicol at 15 or 34 μg/ml, and ampicillin at 100 μg/ml) were tested in LB broths and on LB agar plates (Supplementary Figure S3). We found that the wild-type *S. oneidensis* MR-1 could grow on media containing ampicillin, indicating natural resistance to this antibiotic. Based on antibiotic sensitivity tests, two different genes conferring resistance to chloramphenicol and kanamycin were selected. Next, we addressed the use of the shuttle element *oriT*, an alternative to the *mob* gene, since transformation of *S. oneidensis* MR-1 relies on conjugation. Bacterial conjugation machinery is composed of *RK2-oriT* modules containing the *traJ* gene (Smillie et al., 2010), which is needed within a plasmid to enable its uptake by the recipient cell through conjugation. We introduced the *RK2-oriT* cassette, which is smaller than the *mob* gene, into the vector backbone and demonstrated successful transformation from *E. coli* to *S. oneidensis* MR-1. Using this efficient transfer system, longer genes can be accommodated and expressed in the vectors.

**FIGURE 4:**
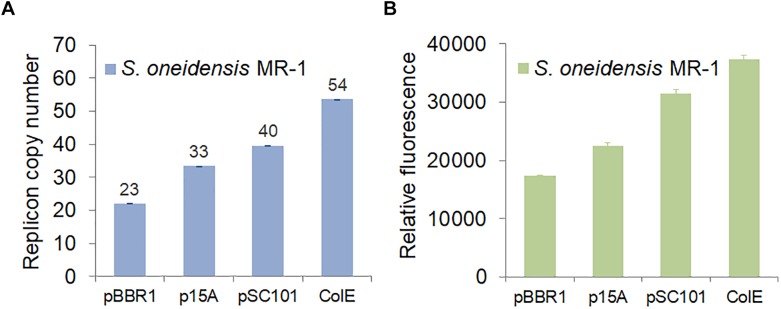
Characterization of replication origins in *S. oneidensis* MR-1. **(A)** Replicon copy numbers of different replication origins in *S. oneidensis* MR-1 determined by RT-qPCR. **(B)** GFP expression of various replication origins in *S. oneidensis* MR-1. *S. oneidensis* MR-1 harbored a single plasmid carrying pBBR1, p15A, pSC101, or ColE replication origin. In each case, the *gfp* gene was expressed from the same promoter, pTrc^∗^ (no repressor lacO). The error bars (mean ± SD) were derived from triplicate experiments for each strain.

In order to be compatible with the commonly used vector pHG101, we used the chloramphenicol resistance (CmR) gene and the shuttle *oriT* gene to test various replication origins. We chose ColE, p15A, and pSC101, which are commonly used in *E. coli*, and repB, which was used as a replication origin in the shuttle vector pSXM33 from *Shewanella xiamenensis* BC01 (Zhou and Ng, 2016). We determined that shuttle vectors carrying ColE, p15A, or pSC101 could be efficiently imported into *S. oneidensis* MR-1. However, *S. oneidensis* MR-1 carrying repB could not grow due to failed transformation into *E. coli* and *S. oneidensis* MR-1. When considering and assessing replication origins, plasmid copy number is a key parameter. Hajimorad and Gralnick (2016) showed that different replication origins (p15A, pMB1, and pBBR1) could be maintained in *Shewanella* by counting colony forming unites (CFU). Here, we determined the copy number of different replicons using real-time quantitative PCR (RT-qPCR). As depicted in Figure 4A, the results indicated that the CoIE replication origin had a copy number of 54, which was higher than the other replication origins: pSC101 (40), p15A (33), and pBBR1 (23). Meanwhile, we also measured the corresponding fluorescence of strains carrying these replication origins (Figure 4B), demonstrating that fluorescence was in accordance with copy number.

**FIGURE 5:**
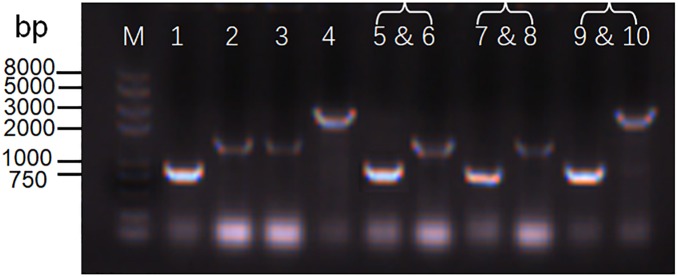
Determination of the coexistence of two plasmids in *S. oneidensis* MR-1. Agarose gel electrophoresis analysis of replication origins from a single plasmid or two plasmids in *S. oneidensis* MR-1. M: Trans2K Plus ll DNA Marker. Lanes 1–4: a single plasmid containing replication origin pBBR1 (Lane 1: 840 bp), ColE (Lane 2: 1,145 bp), p15A (Lane 3: 1,132 bp), and pSC101 (Lane 4: 2,535 bp). Lanes 5–10: three groups of two plasmids containing replication origins pBBR1/ColE (Lanes 5 and 6), pBBR1/p15A (Lanes 7 and Lane 8), and pBBR1/pSC101 (Lanes 9 and 10).

While screening the replication origins, we also tested whether the origins ColE, p15A, and pSC101 were compatible with the commonly used replication origin, pBBR1, in *S. oneidensis* MR-1. We co-transformed *S. oneidensis* MR-1 with two vectors carrying different replication origins: pBBR1/ColE, pBBR1/p15A, and pBBR1/pSC101. Plasmids carrying the pBBR1 origin confer kanamycin resistance, while plasmids carrying the ColE, p15A, or pSC101 origin confer chloramphenicol resistance. Transfer and maintenance of either a single plasmid or two plasmids (containing two replication origins) in *S. oneidensis* MR-1 were verified by colony PCR (Figure 5) using primers listed in Supplementary Table S1. The expected lengths of PCR products were 840 bp, 1,145 bp, 1,132 bp, and 2,535 bp for *pBBR1*, *CoIE, p15A*, and *pSC101*, respectively. Agarose gel electrophoresis analysis confirmed band sizes, as shown from Lane 1 to Lane 4 in Figure 5. Lanes 5–10 depict three groups of two-plasmid containing replication origins: pBBR1/ColE (Lanes 5 and 6), pBBR1/p15A (Lanes 7 and 8), and pBBR1/pSC101 (Lanes 9 and 10). The results revealed compatibility of ColE/pBBR1, p15A/pBBR1, and pSC101/pBBR1 as well as successful co-transformation and maintenance of multiple plasmids in *S. oneidensis* MR-1.

### Fine-Tuning the Expression of Multiple Fluorescent Proteins in *S. oneidensis*

The above results demonstrated the utility of the developed plasmid toolkit, which enabled precise control of gene expression using different strength promoters, regulating the inducer concentration or induction time for inducible promoters, and using various replication origins with different copy numbers. Fine-tuning gene expression can be achieved by adopting suitable elements with different levels of expression according to the actual experimental needs. The next step was to test the ability of the modular plasmids to modulate the simultaneous expression of multiple genes in *S. oneidensis* MR-1. For this purpose, three fluorescent proteins (i.e., GFP, BFP, and CFP) were chosen and expressed in separated plasmids.

**FIGURE 6:**
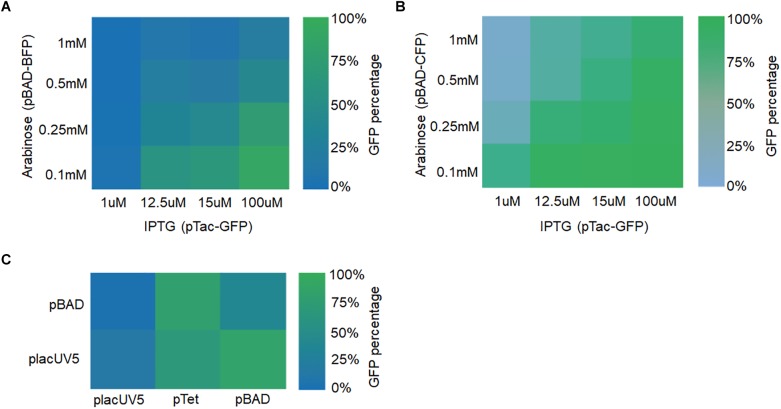
Fine-tuning multiple fluorescent protein expression in *S. oneidensis* MR-1 harboring two plasmids with replicons pBBR1 and CoIE. **(A)** GFP and BFP expression were regulated by the IPTG-inducible pTac promoter (IPTG concentrations: 1, 12.5, 15, and 100 μM) and the arabinose-inducible pBAD promoter (arabinose concentrations: 0.1, 0.25, 0.5, and 1 mM), respectively. **(B)** GFP and CFP expression were adjusted by the promoters pTac and pBAD at the above concentrations, respectively. **(C)** GFP and BFP expression were under the control of placUV5, pTet, or pBAD, and placUV5 or pBAD, respectively. The error bars (mean ± SD) were derived from triplicate experiments for each strain.

Two manipulation strategies were applied. Firstly, two different fluorescent genes were expressed using different types of promoters (e.g., IPTG-inducible or arabinose-inducible) with two-gene expression fine-tuned by adjusting the inducer concentrations. GFP was expressed using vectors with the IPTG-inducible promoter pTac, the pBBR1 replication origin, and the kanamycin resistance (Kan) gene. The following IPTG concentrations were assessed: 1, 12.5, 15, and 100 μM. BFP was expressed using vectors with the arabinose-inducible promoter pBAD, the ColE replication origin, and the chloramphenicol resistance (CmR) gene. The following arabinose concentrations were used: 0.1, 0.25, 0.5, and 1 mM. As shown in Figure 6A and Supplementary Figure S4, higher GFP fluorescence was observed for higher concentrations of IPTG and lower concentrations of arabinose. Likewise, higher BFP fluorescence was observed for higher concentrations of arabinose and lower concentrations of IPTG. Furthermore, similar results were obtained when BFP was replaced with CFP (Figure 6B). The only difference was that CFP expression level was relatively low (Supplementary Figure S5). In all, these results demonstrated that the expression of different genes in *S. oneidensis* MR-1 could be obtained by adjusting inducer concentrations for the inducible promoters.

Secondly, fine-tuning of gene expression was achieved by utilizing different strength promoters (low, medium, and high) at their normal induction concentrations. GFP was expressed under the control of placUV5, pTet, and pBAD promoters on a plasmid containing the pBBR1 replication origin and the kanamycin resistance (Kan) gene. BFP was expressed under the control of placUV5 and pBAD promoters on a separate plasmid containing the ColE replication origin and the chloramphenicol resistance (CmR) gene. We co-transformed the two plasmid types into *S. oneidensis* MR-1. As depicted in Figure 6C and Supplementary Figure S6, when the GFP was expressed under the control of the high-strength pBAD promoter and BFP was expressed under the control of the low-strength placUV5 promoter, high GFP and low BFP were detected. Similarly, when the BFP was expressed under the control of the high-strength pBAD promoter and GFP was expressed under the control of the low-strength placUV5 promoter, low GFP and high BFP were detected. In all, different levels of gene expression could also be achieved by using promoters of different strengths. Overall, the plasmid toolkit developed here can be used to delicately fine-tune the simultaneous expression of multiple genes using different strategies in *S. oneidensis*.

### Fine-Tuning MtrCAB Expression in the *S. oneidensis* Δ*mtrA* Strain

The EET pathway of *S. oneidensis* MR-1 is comprised of *c*-type cytochromes that shuttle electrons from oxidizing enzymes in the cytoplasm and inner membrane to the outside of the cell during anaerobic incubation (Schuetz et al., 2009; Dan et al., 2010; Gralnick and Newman, 2010; Ross et al., 2012). The *c*-type cytochrome MtrCAB complex is regarded as the primary route of EET and a potentially minimal set. In *E. coli*, heterologous expression and regulation of the genes in the Mtr pathway (Gescher et al., 2010; Jensen et al., 2010; Goldbeck et al., 2013) suggest that too much expression of MtrCAB could cause pleotropic impairments in the host organism and diminish EET efficiency. Thus, MtrCAB expression should be delicately fine-tuned to improve EET capacity.

**FIGURE 7:**
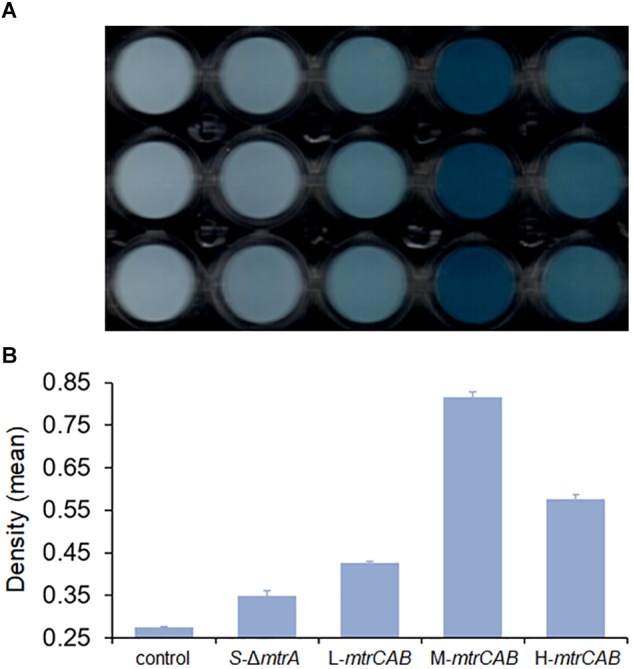
Fine-tuning MtrCAB expression to affect EET efficiency determined by WO_3_ reduction assay. **(A)** Color development of the WO_3_ reduction assay. Culture medium without bacteria was used as a control to eliminate disturbances caused by abiotic factors. The *mtrCAB* gene was overexpressed in the *S. oneidensis* Δ*mtrA* strain under the control of promoters placUV5, pTet, and pBAD, which were named as *S-*Δ*mtrA*, L-*mtrCAB*, M-*mtrCAB*, and H-*mtrCAB*, respectively, according to their level of expression (L, low; M, medium; H, high). **(B)** ‘Density (mean)’ analysis of each strain from the WO_3_ reduction assay using ImageJ Software V.1.8.0. The error bars (mean ± SD) were derived from triplicate experiments for each strain.

To illustrate whether the plasmid toolkit could be applied in EET manipulation in *S. oneidensis*, the *mtrCAB* was taken as an example to be overexpressed. The *mtrCAB* gene cluster was cloned from the *S. oneidensis* genome and overexpressed in the *S. oneidensis* Δ*mtrA* strain under the control of placUV5, pTet, and pBAD promoters, which displayed low, medium, and high levels of gene expression, respectively. The parental and recombinant strains were named as *S-*Δ*mtrA*, L-*mtrCAB*, M-*mtrCAB*, and H-*mtrCAB*, respectively. Firstly, we performed a tungsten trioxide (WO_3_) reduction assay (Yuan et al., 2014) to assess the fine-tuning effect of *mtrCAB* on EET. Culture medium without bacteria was used as a control to eliminate disturbances caused by abiotic factors. EET from *S-*Δ*mtrA*, L-*mtrCAB*, M-*mtrCAB*, or H-*mtrCAB* to the WO_3_ nanorod assembly probe was accompanied by a bioelectrochromic reaction causing conspicuous color development in the 96-well plate, which enabled us to evaluate EET either qualitatively with the naked eye or quantitatively through image analysis (Figure 7A). For the latter, the color intensity of each well was determined by analyzing the ‘Density (mean)’ (Wang et al., 2009; Tatro et al., 2013) of the area of interest with ImageJ Software V.1.8.0 (Figure 7B). The results showed that the EET capacity of MtrCAB under the control of the low-, medium-, and high-strength promoters was increased by 22.3, 133.5, and 65.3% over that of the parental strain, suggesting that overexpression of MtrCAB at a medium level improved EET most significantly in the *S. oneidensis* Δ*mtrA* strain.

**FIGURE 8:**
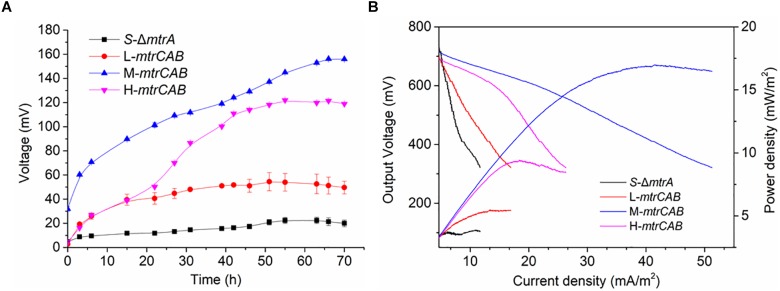
Fine-tuning MtrCAB expression to affect EET efficiency determined by MFC assay. **(A)** Voltage output of the *S-*Δ*mtrA*, L-*mtrCAB*, M-*mtrCAB*, and H-*mtrCAB* strains in MFCs. **(B)** MFC polarization curves and power density output curves obtained by linear sweep voltammetry (LSV) with a scan rate of 0.1 mV/s. The error bars (mean ± SD) were derived from triplicate experiments for each strain.

To more quantitatively investigate EET efficiency upon fine-tuning the overexpression of MtrCAB, bio-electrochemical analyses were conducted in microbial fuel cells (MFCs). The parental and engineered strains were inoculated into the anodic chamber of MFCs, respectively, with a 2 kΩ external resistor (Figure 8). Experimental results showed that MtrCAB expression in the mutant *S. oneidensis* could increase the voltage output, with significant differences among the recombinant strains. The maximum voltage increased 140.3, 595.8, and 443.2%, when the *mtrCAB* complex was expressed at low, medium and high levels, respectively, compared to the parental *S. oneidensis*Δ*mtrA* strain (Figure 8A). Bioelectrochemical analyses were further conducted to study EET efficiency of these recombinant strains in MFCs (Figure 8B). Polarization discharge curves and power density output curves were obtained by linear sweep voltammetry (LSV) with a 0.1 mV/s scan rate. The dropping slope of the polarization curve obtained from M-*mtrCAB* (shown in the blue curve) was smaller than that of the other two recombinant strains (L-*mtrCAB* shown in red; and H-*mtrCAB*, shown in magenta), implying that the internal charge transfer resistance of the MFC inoculated with M-*mtrCAB* was relatively smaller. Furthermore, the power density output curves showed that the M-*mtrCAB* strain obtained a maximum power density of ∼17.0 mW/m^2^, which was ∼1.8, ∼3.1, and ∼4.5 times higher than that of H-*mtrCAB*, L-*mtrCAB*, and *S-ΔmtrA*, respectively (Figure 8B). These MFC results demonstrated that EET efficiency was improved most significantly when *mtrCAB* was expressed using a moderate promoter in the mutant *S. oneidensis*, which was consistent with the WO_3_ assay analyses. These results validate that the plasmid toolkit we developed could indeed be applied to manipulate EET and metabolism in *S. oneidensis*.

## Conclusion

We constructed and characterized a synthetic plasmid toolkit for *S. oneidensis* MR-1, which contains a set of shuttle vectors with a combination of promoters, replication origins, antibiotic resistance genes, and a shuttle component. We quantified the strength of promoters and the copy number of different replicons. We further transformed two plasmids conferring different antibiotic resistances into recombinant *S. oneidensis* MR-1 strains, enabling the expression of multiple genes (e.g., fluorescent reporters) to be fine-tuned. The synthetic plasmid toolkit was then successfully used to modulate the expression of MtrCAB and achieve improved EET efficiency. Moderate overexpression level of MtrCAB improved the EET efficiency most significantly in the *S. oneidensis* Δ*mtrA* strain. The plasmid toolkit developed in this study allows researchers to rapidly and conveniently fine-tune gene expressions with greater controllability and predictability, thus accelerating the development and application of genetic manipulations in *S. oneidensis*.

## Data Availability

This manuscript contains previously unpublished data. The name of the repository and accession number are not available.

## Author Contributions

Y-XC designed the experiments. M-YS and Y-XC performed all the experiments. Y-RC, Y-YC, JX, and QD performed a part of MFC experiments. FL, C-FL, and XL helped with revising the manuscript. M-YS, Y-XC, and HS wrote the manuscript.

## Conflict of Interest Statement

The authors declare that the research was conducted in the absence of any commercial or financial relationships that could be construed as a potential conflict of interest.
