# Supplementary material for: A Synthetic Plasmid Toolkit for Shewanella oneidensis MR-1
Source: Front Microbiol. 2019 Mar 8;10:410. doi: 10.3389/fmicb.2019.00410 (PMC6418347; doi:10.3389/fmicb.2019.00410)
Supplement: Supplementary file 1 [file Table_1.docx]

***Supplementary Material***

**A Synthetic Plasmid Toolkit for *Shewanella oneidensis* MR-1**

Yingxiu Cao^1^, Mengyuan Song^1^, Feng Li^1^, Congfa Li^2^, Xue Lin^2^, Yaru Chen^1^, Yuanyuan Chen^1^, Jing Xu^1^, Qian Ding^1^, Hao Song^1,^ *

^1^ Key Laboratory of Systems Bioengineering (Ministry of Education), SynBio Research Platform, Collaborative Innovation Center of Chemical Science and Engineering (Tianjin), and School of Chemical Engineering and Technology, Tianjin University, Tianjin, 300072, P. R. China.

^2^ College of Food Science and Technology, Hainan University, Haikou 570228, P. R. China.

Ying-Xiu Cao, E-mail: [caoyingxiu@tju.edu.cn](mailto:caoyingxiu@tju.edu.cn);

*Corresponding author: Hao Song, E-mail: [hsong@tju.edu.cn](mailto:hsong@tju.edu.cn)

Supporting Figures


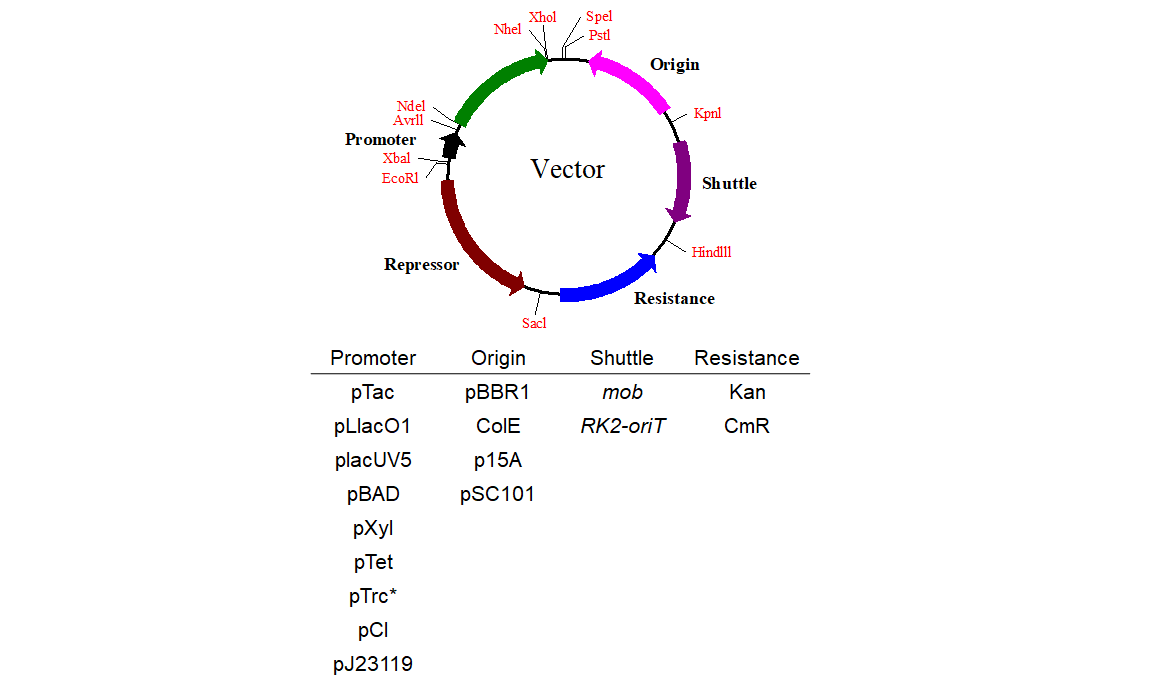


FIGURE S1 | The plasmid toolkit design and construction. The toolkit is composed of four modules: replication origin module, antibiotic resistance gene module, shuttle module, and expression module, which includes the promoter, repressor, and the gene of interest. These modules could be easily replaced by relative restriction enzyme sites, which are presented in red. The repressor means lacI for pTac, pLlacO1, placUV5 promoters in this work. The repressor araC, xylR, or tetR and its corresponding promoter pBAD, pXyl, or pTet are connected together and inserted after the Xbal site as one part.


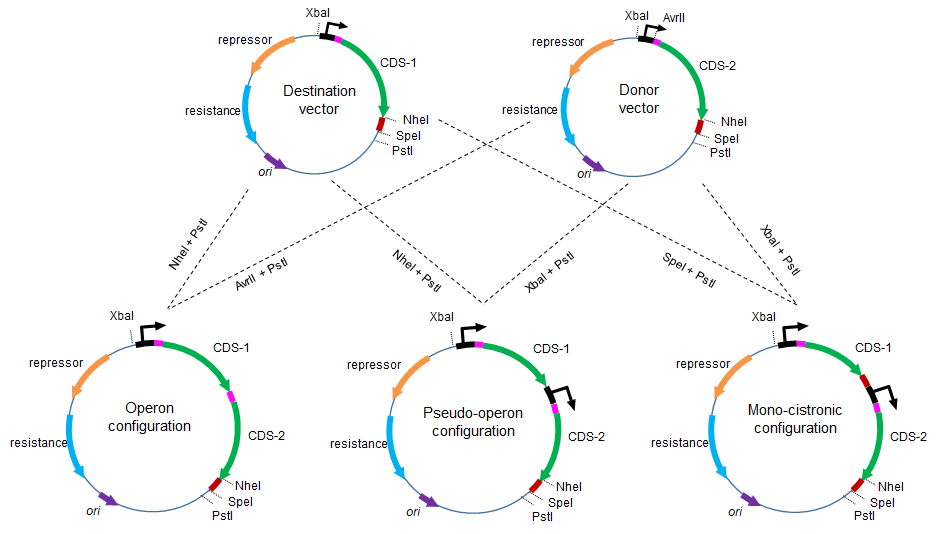


**FIGURE S2 |** Expression cassettes are designed as BioBrick standard for versatile genes construction. The BioBrick vectors feature in four isocaudamer pairs (Avrll, Ndel, XbaI, and SpeI) can form the monocistron or polycistron, and support the modular assembly of numbers of molecular components and multigene pathways.


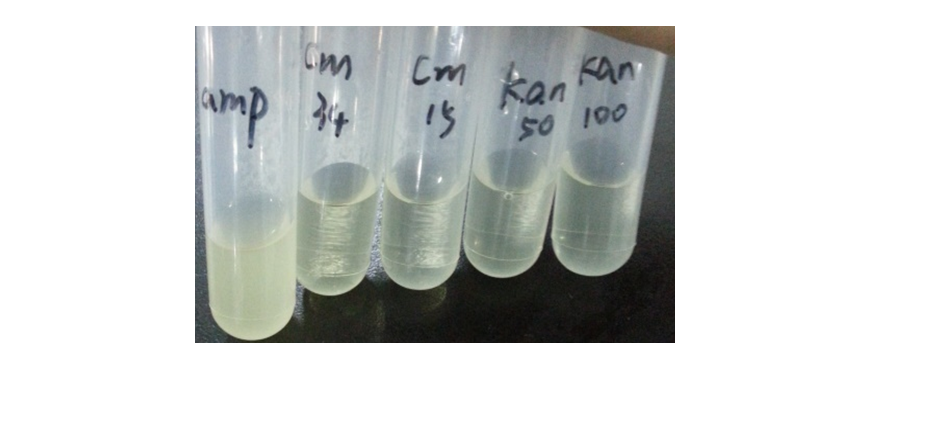


FIGURE S3 | Characterization of antibiotic resistance genes (Kan, CmR, Amp) in *S. oneidensis* MR-1 at 30℃. Wild *S. oneidensis* MR-1 carried Kan, CmR, Amp resistance genes in LB brothes at 30℃.


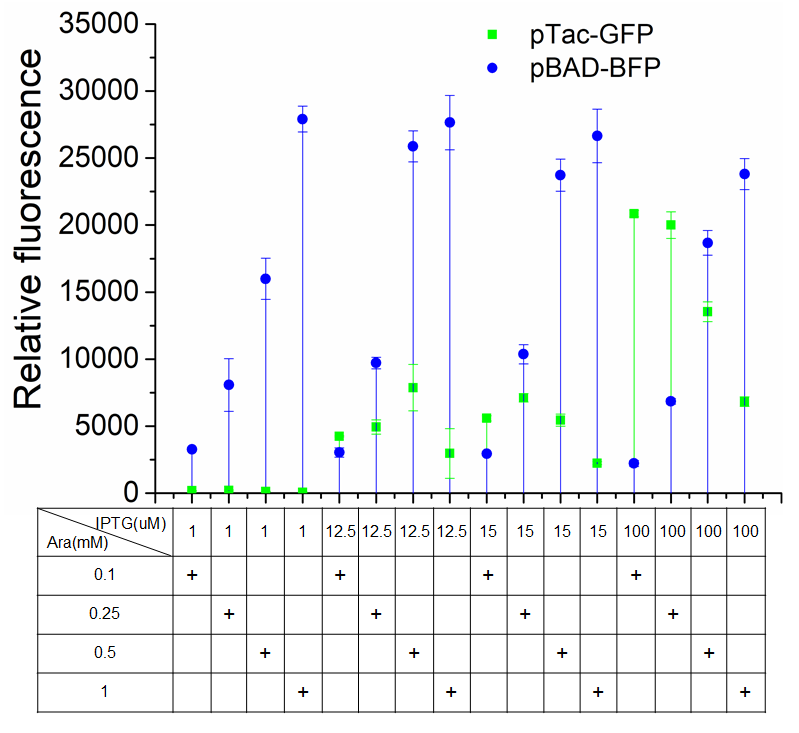


FIGURE S4 | Relative fluorescences of GFP and BFP in the fine-tuning experiment (FIGURE 6A). GFP was under the control of IPTG-inducible promoter pTac and BFP was under the control of arabinose-inducible promoter pBAD. The error bars (mean±SD) were derived from triplicate experiments for each strain.


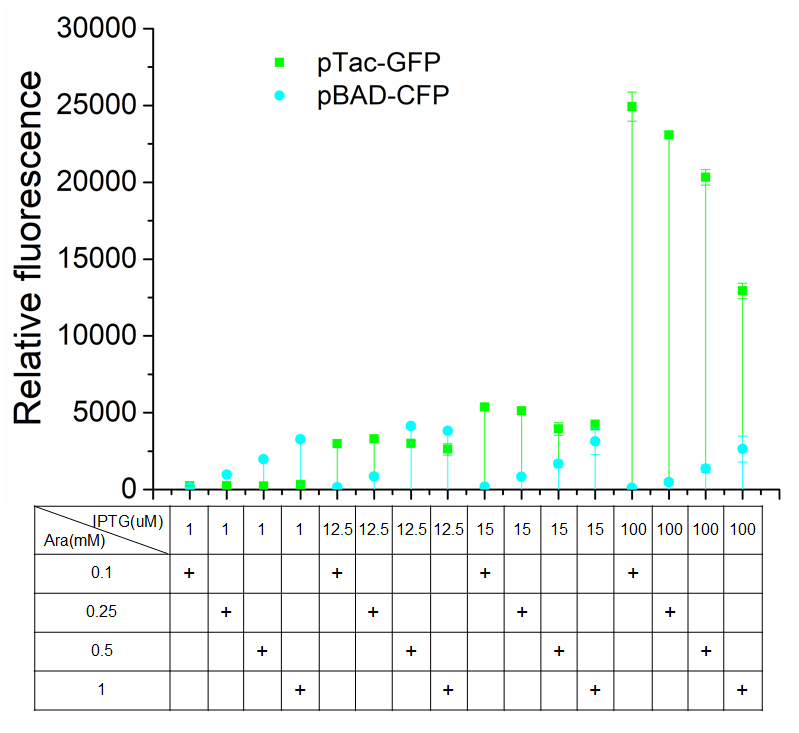


FIGURE S5 | Relative fluorescence of GFP and CFP in the fine-tuning experiment (FIGURE 6B). GFP was under the control of IPTG-inducible promoter pTac and CFP was under the control of arabinose-inducible promoter pBAD. The error bars (mean±SD) were derived from triplicate experiments for each strain.


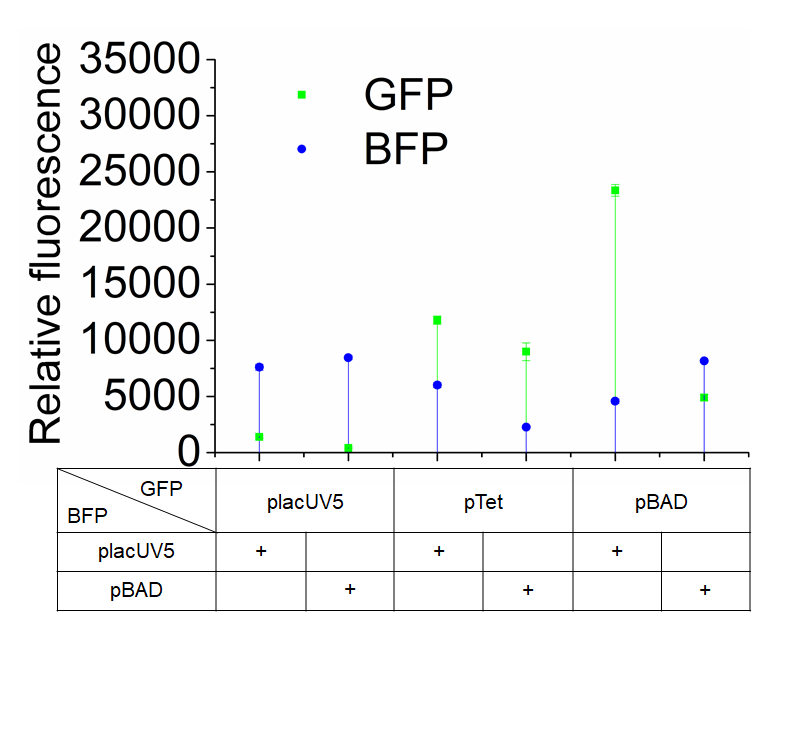


FIGURE S6 | Relative fluorescence of GFP and BFP in the fine-tuning experiment (FIGURE 6C). GFP was under the control of placUV5, pTet, pBAD, and BFP was under the control of placUV5, pBAD, respectively. The error bars (mean±SD) were derived from triplicate experiments for each strain.

Supporting Tables

Table S1. Primer sequences

| Primers for pHG12 construction | |
| --- | --- |
| pHG12-ΔNcoI-F | GTGGGTCACGACGAGATCCTC |
| pHG12-ΔNcoI-R | AGGATCTCGTCGTGACCCACGGCGATGCCTGCTTGCCGAATAT |
| SacI-pHG12-F | GTAGTCAATAAACCGGTTGAGCTCAGAGAATTCGCCTCTGCAGACCGG |
| SacI-pHG12-R | TCTGAGCTCAACCGGTTTATTGACTACCG |
| Primers for *mtrCAB* expression vectors construction | |
| MtrCAB-F | CGGCACATATGATGAACGCACAAAAA |
| MtrCAB-R | TACCCTCGAGCCGCTAGCTTAGAGTTTGTAACTCATGCT |
| Primers for standards for qPCR construction | |
| CoIE-F | GCGAAACCCGACAGGACT |
| CoIE-R | CGAACGACCTACACCGAACT |
| p15A-F | CATCACGAAATCTGACGCTC |
| p15-R | GTAAACCGAAAGGCAGGAAC |
| pSC101-F | TCCTGATTTCCACAGTTCTCG |
| pSC101-R | TGCTGTGTGGCACTACTCAAC |
| pBBR1-F | AGGACGAATCGCTGTTGG |
| pBBR1-R | CCGTTTCGGTCAAGGCT |
| Primers for detection of replication origins by colony PCR | |
| pHG12-pBBR1-F | CAACGCATAATTGTTGTCGC |
| pHG12-pBBR1-R | GGAGGCAGACAAGGTATAGGG |
| pHG13-origin-F | AAACGAAAGGCTCAGTCGA |
| pHG13-origin-R | AGCAGGATTCCCGTTGAG |

Table S2. Sequences of codon-optimized genes

Red, blue, yellow sequence is promoter, RBS and terminator sequences, respectively. Start and stop codon are shown in italic.

SacI: GAGCTC; EcoRI: GAATTC; XbaI: TCTAGA; AvrII: CCTAGG; NdeI: CATATG; NheI: GCTAGC; XhoI: CTCGAG; SpeI: ACTAGT; PstI: CTGCAG; KpnI: GGTACC; HindIII: AAGCTT

*>pBAD*

GAATTCGCGGCCGCTTCTAGAGCCTGTCAAATGGACGAAGCAGGGATTCTGCAAACCCTATGCTACTCCGTCAAGCCGTCAATTGTCTGATTCGTTACCAATTATGACAACTTGACGGCTACATCATTCACTTTTTCTTCACAACCGGCACGGAACTCGCTCGGGCTGGCCCCGGTGCATTTTTTAAATACCCGCGAGAAATAGAGTTGATCGTCAAAACCAACATTGCGACCGACGGTGGCGATAGGCATCCGGGTGGTGCTCAAAAGCAGCTTCGCCTGGCTGATACGTTGGTCCTCGCGCCAGCTTAAGACGCTAATCCCTAACTGCTGGCGGAAAAGATGTGACAGACGCGACGGCGACAAGCAAACATGCTGTGCGACGCTGGCGATATCAAAATTGCTGTCTGCCAGGTGATCGCTGATGTACTGACAAGCCTCGCGTACCCGATTATCCATCGGTGGATGGAGCGACTCGTTAATCGCTTCCATGCGCCGCAGTAACAATTGCTCAAGCAGATTTATCGCCAGCAGCTCCGAATAGCGCCCTTCCCCTTGCCCGGCGTTAATGATTTGCCCAAACAGGTCGCTGAAATGCGGCTGGTGCGCTTCATCCGGGCGAAAGAACCCCGTATTGGCAAATATTGACGGCCAGTTAAGCCATTCATGCCAGTAGGCGCGCGGACGAAAGTAAACCCACTGGTGATACCATTCGCGAGCCTCCGGATGACGACCGTAGTGATGAATCTCTCCTGGCGGGAACAGCAAAATATCACCCGGTCGGCAAACAAATTCTCGTCCCTGATTTTTCACCACCCCCTGACCGCGAATGGTGAGATTGAGAATATAACCTTTCATTCCCAGCGGTCGGTCGATAAAAAAATCGAGATAACCGTTGGCCTCAATCGGCGTTAAACCCGCCACCAGATGGGCATTAAACGAGTATCCCGGCAGCAGGGGATCATTTTGCGCTTCAGCCATACTTTTCATACTCCCGCCATTCAGAGAAGAAACCAATTGTCCATATTGCATCAGACATTGCCGTCACTGCGTCTTTTACTGGCTCTTCTCGCTAACCAAACCGGTAACCCCGCTTATTAAAAGCATTCTGTAACAAAGCGGGACCAAAGCCATGACAAAAACGCGTAACAAAAGTGTCTATAATCACGGCAGAAAAGTCCACATTGATTATTTGCACGGCGTCACACTTTGCTATGCCATAGCATTTTTATCCATAAGATTAGCGGATCCTACCTGACGCTTTTTATCGCAACTCTCTACTGTTTCTCCATACCCGTTTTTTTGGGCCCTAGGAATAATTTTGTTTAACTTTAAGAAGGAGATATACAT*ATG*TAAGCTAGCAGCTCGAGCAAATAAAACGAAAGGCTCAGTCGAAAGACTGGGCCTTTCGTTTTATCTGTTGTTTGTCGGTGAACGCTCTCCTGAGTAGGACAAATTACTAGTAGCGGCCGCTGCAG

*>pXyl*

GAATTCGCGGCCGCTTCTAGAGCTAACTTATAGGGGTAACACTTAAAAAAGAATCAATAACGATAGAAACCGCTCCTAAAGCAGGTGCATTTTTTCCTAACGAAGAAGGCAATAGTTCACATTTATTGTCTAAATGAGAATGGACTCTCGAAGAAACTTCGTTTTTAATCGTATTTAAAACAATGGGATGAGATTCAATTATATGATTTCTCAAGATAACAGCTTCTATATCAAATGTATTAAGGATATTGGTTAATCCAATTCCGATATAAAAGCCAAAGTTTTGAAGTGCATTTAACATTTCTACATCATTTTTATTTGCGCGTTCCACAATCTCTTTTCGAGAAATATTCTTTTCTTCTTTAGAGAGCGAAGCCAGTAACGCTTTTTCAGAAGCATATAATTCCCAACAGCCTCGATTTCCACAGCTGCATTTGGGTCCATTAAAATCTATCGTCATGTGACCCATTTCCCCAGAAAAACCCTGAACACCTTTATACAATTCGTTGTTAATAACAAGTCCAGTTCCAATTCCGATATTAATACTGATGTAAACGATGTTTTCATAGTTTTTTGTCATACCAAATACTTTTTCACCGTATGCTCCTGCATTAGCTTCATTTTCAACAAAAACCGGAACATTAAACTCACTCTCAATTAAAAACTGCAAATCTTTGATATTCCAATTTAAGTTAGGCATGAAAATAATTTGCTGATGACGATCTACAAGGCCTGGAACACAAATTCCTATTCCGACTAGACCATAAGGGGACTCAGGCATGTGGGTTACAAAACCATGAATAAGTGCAAATAAAATCTCTTTTACTTCACTAGCGGAAGAACTAGACAAGTCAGAAGTCTTCTCCAGAATAATATTTCCTTCTAAGTCGGTTAGAATGCCGTTAAGATAGTCGACTCCTATATCAATACCAATCGAGTAGCCTGCATTCTTATTAAAAACAAGCATTACAGGTCTTCTGCCGCCTCTCGATTGCCCTGCCCCAATTTCAAAAATAAAATCTTTTTCAAGCAGTGTATTTACTTGAGAGGAGACAGTAGACTTGTTTAATCCTGTAATCTCAGAGAGAGTTGCCCTGGAGACAGGGGAGTTCTTCAAAATTTCATCTAATATTAATTTTTGATTCATTTTTTTTACTAAAGCTTGATCTGCAATTTGAATAATAACCACTCCTTTGTTTATCCACCGAACTAAGTTGGTGTTTTTTGAAGCTTGAATTAGATATTTAAAAGTATCATATCTAATATTATAACTAAATTTTCTAAAAAAAACATTGAAATAAACATTTATTTTGTATATGATGAGATAAAGTTAGTTTATTGGATAAACAAACTAACTCAATTAAGATAGTTGATGGATAAACTTGTTCACTTAAATCAAAGGGGGAAATGACAT*ATG*TAAGCTAGCAGCTCGAGCAAATAAAACGAAAGGCTCAGTCGAAAGACTGGGCCTTTCGTTTTATCTGTTGTTTGTCGGTGAACGCTCTCCTGAGTAGGACAAATTACTAGTAGCGGCCGCTGCAG

*>pTet*

GAATTCGCGGCCGCTTCTAGAGTCTTAAGACCCACTTTCACATTTAAGTTGTTTTTCTAATCCGCAAATGATCAATTCAAGGCCGAATAAGAAGGCTGGCTCTGCACCTTGGTGATCAAATAATTCGATAGCTTGTCGTAATAATGGCGGCATACTATCAGTAGTAGGTGTTTCCCTTTCTTCTTTAGCGACTTGATGCTCTTGATCTTCCAATACGCAACCTAAAGTAAAATGCCCCACAGCGCTGAGTGCATATAATGCATTCTCTAGTGAAAAACCTTGTTGGCATAAAAAGGCTAATTGATTTTCGAGAGTTTCATACTGTTTTTCTGTAGGCCGTGTACCTAAATGTACTTTTGCTCCATCGCGATGACTTAGTAAAGCACATCTAAAACTTTTAGCGTTATTACGTAAAAAATCTTGCCAGCTTTCCCCTTCTAAAGGGCAAAAGTGAGTATGGTGCCTATCTAACATCTCAATGGCTAAGGCGTCGAGCAAAGCCCGCTTATTTTTTACATGCCAATACAATGTAGGCTGCTCTACACCTAGCTTCTGGGCGAGTTTACGGGTTGTTAAACCTTCGATTCCGACCTCATTAAGCAGCTCTAATGCGCTGTTAATCACTTTACTTTTATCTAATCTGCTCATCATTAATTCCTAATTTTTGTTGACACTCTATCGTTGATAGAGTTATTTTACCACTCCCTATCAGTGATAGAGAAAAGCCTAGGAATAATTTTGTTTAACTTTAAGAAGGAGATATACAT*ATG*TAAGCTAGCAGCTCGAGCAAATAAAACGAAAGGCTCAGTCGAAAGACTGGGCCTTTCGTTTTATCTGTTGTTTGTCGGTGAACGCTCTCCTGAGTAGGACAAATTACTAGTAGCGGCCGCTGCAG

*>pTrc**

GAATTCGCGGCCGCTTCTAGAGAAATATTCTGAAATGAGCTGTTGACAATTAATCATCCGGCTCGTATAATGTGTGGAACCTAGGAATAATTTTGTTTAACTTTAAGAAGGAGATATACAT*ATG*TAAGCTAGCAGCTCGAGCAAATAAAACGAAAGGCTCAGTCGAAAGACTGGGCCTTTCGTTTTATCTGTTGTTTGTCGGTGAACGCTCTCCTGAGTAGGACAAATTACTAGTAGCGGCCGCTGCAG

*>pTac*

GAGCTCGCCAGAACCGTTATGATGTCGGCGCAAAAAACATTATCCAGAACGGGAGTGCGCCTTGAGCGACACGAATTATGCAGTGATTTACGACCTGCACAGCCATACCACAGCTTCCGATGGCTGCCTGACGCCAGAAGCATTGGTGCACCGTGCAGTCGATGATAAGCTGTCAAACATGAGAATTGTGCCTAATGAGTGAGCTAACTTACATTAATTGCGTTGCGCTCACTGCCCGCTTTCCAGTCGGGAAACCTGTCGTGCCAGCTGCATTAATGAATCGGCCAACGCGCGGGGAGAGGCGGTTTGCGTATTGGGCGCCAGGGTGGTTTTTCTTTTCACCAGTGAGACGGGCAACAGCTGATTGCCCTTCACCGCCTGGCCCTGAGAGAGTTGCAGCAAGCGGTCCACGCTGGTTTGCCCCAGCAGGCGAAAATCCTGTTTGATGGTGGTTAACGGCGGGATATAACATGAGCTGTCTTCGGTATCGTCGTATCCCACTACCGAGATATCCGCACCAACGCGCAGCCCGGACTCGGTAATGGCGCGCATTGCGCCCAGCGCCATCTGATCGTTGGCAACCAGCATCGCAGTGGGAACGATGCCCTCATTCAGCATTTGCATGGTTTGTTGAAAACCGGACATGGCACTCCAGTCGCCTTCCCGTTCCGCTATCGGCTGAATTTGATTGCGAGTGAGATATTTATGCCAGCCAGCCAGACGCAGACGCGCCGAGACAGAACTTAATGGGCCCGCTAACAGCGCGATTTGCTGGTGACCCAATGCGACCAGATGCTCCACGCCCAGTCGCGTACCGTCTTCATGGGAGAAAATAATACTGTTGATGGGTGTCTGGTCAGAGACATCAAGAAATAACGCCGGAACATTAGTGCAGGCAGCTTCCACAGCAATGGCATCCTGGTCATCCAGCGGATAGTTAATGATCAGCCCACTGACGCGTTGCGCGAGAAGATTGTGCACCGCCGCTTTACAGGCTTCGACGCCGCTTCGTTCTACCATCGACACCACCACGCTGGCACCCAGTTGATCGGCGCGAGATTTAATCGCCGCGACAATTTGCGACGGCGCGTGCAGGGCCAGACTGGAGGTGGCAACGCCAATCAGCAACGACTGTTTGCCCGCCAGTTGTTGTGCCACGCGGTTGGGAATGTAATTCAGCTCCGCCATCGCCGCTTCCACTTTTTCCCGCGTTTTCGCAGAAACGTGGCTGGCCTGGTTCACCACGCGGGAAACGGTCTGATAAGAGACACCGGCATACTCTGCGACATCGTATAACGTTACTGGTTTCACATTCACCACCCTGAATTGACTCTCTTCCGGGCGCTATCATGCCATACCGCGAAAGGTTTTGCACCATTCGATGGTGTCGAATTCGCGGCCGCTTCTAGAGAAATATTCTGAAATGAGCTGTTGACAATTAATCATCGGCTCGTATAATGTGTGGAATTGTGAGCGGATAACAATTCCTAGGAATAATTTTGTTTAACTTTAAGAAGGAGATATACAT*ATG*TAAGCTAGCAGCTCGAGCAAATAAAACGAAAGGCTCAGTCGAAAGACTGGGCCTTTCGTTTTATCTGTTGTTTGTCGGTGAACGCTCTCCTGAGTAGGACAAATTACTAGTAGCGGCCGCTGCAG

*>pLlacO1*

GAGCTCGCCAGAACCGTTATGATGTCGGCGCAAAAAACATTATCCAGAACGGGAGTGCGCCTTGAGCGACACGAATTATGCAGTGATTTACGACCTGCACAGCCATACCACAGCTTCCGATGGCTGCCTGACGCCAGAAGCATTGGTGCACCGTGCAGTCGATGATAAGCTGTCAAACATGAGAATTGTGCCTAATGAGTGAGCTAACTTACATTAATTGCGTTGCGCTCACTGCCCGCTTTCCAGTCGGGAAACCTGTCGTGCCAGCTGCATTAATGAATCGGCCAACGCGCGGGGAGAGGCGGTTTGCGTATTGGGCGCCAGGGTGGTTTTTCTTTTCACCAGTGAGACGGGCAACAGCTGATTGCCCTTCACCGCCTGGCCCTGAGAGAGTTGCAGCAAGCGGTCCACGCTGGTTTGCCCCAGCAGGCGAAAATCCTGTTTGATGGTGGTTAACGGCGGGATATAACATGAGCTGTCTTCGGTATCGTCGTATCCCACTACCGAGATATCCGCACCAACGCGCAGCCCGGACTCGGTAATGGCGCGCATTGCGCCCAGCGCCATCTGATCGTTGGCAACCAGCATCGCAGTGGGAACGATGCCCTCATTCAGCATTTGCATGGTTTGTTGAAAACCGGACATGGCACTCCAGTCGCCTTCCCGTTCCGCTATCGGCTGAATTTGATTGCGAGTGAGATATTTATGCCAGCCAGCCAGACGCAGACGCGCCGAGACAGAACTTAATGGGCCCGCTAACAGCGCGATTTGCTGGTGACCCAATGCGACCAGATGCTCCACGCCCAGTCGCGTACCGTCTTCATGGGAGAAAATAATACTGTTGATGGGTGTCTGGTCAGAGACATCAAGAAATAACGCCGGAACATTAGTGCAGGCAGCTTCCACAGCAATGGCATCCTGGTCATCCAGCGGATAGTTAATGATCAGCCCACTGACGCGTTGCGCGAGAAGATTGTGCACCGCCGCTTTACAGGCTTCGACGCCGCTTCGTTCTACCATCGACACCACCACGCTGGCACCCAGTTGATCGGCGCGAGATTTAATCGCCGCGACAATTTGCGACGGCGCGTGCAGGGCCAGACTGGAGGTGGCAACGCCAATCAGCAACGACTGTTTGCCCGCCAGTTGTTGTGCCACGCGGTTGGGAATGTAATTCAGCTCCGCCATCGCCGCTTCCACTTTTTCCCGCGTTTTCGCAGAAACGTGGCTGGCCTGGTTCACCACGCGGGAAACGGTCTGATAAGAGACACCGGCATACTCTGCGACATCGTATAACGTTACTGGTTTCACATTCACCACCCTGAATTGACTCTCTTCCGGGCGCTATCATGCCATACCGCGAAAGGTTTTGCACCATTCGATGGTGTCGAATTCGCGGCCGCTTCTAGAGAATTGTGAGCGGATAACAATTGACATTGTGAGCGGATAACAAGATACTGAGCACACCTAGGAATAATTTTGTTTAACTTTAAGAAGGAGATATACAT*ATG*TAAGCTAGCAGCTCGAGCAAATAAAACGAAAGGCTCAGTCGAAAGACTGGGCCTTTCGTTTTATCTGTTGTTTGTCGGTGAACGCTCTCCTGAGTAGGACAAATTACTAGTAGCGGCCGCTGCAG

*>placUV5*

GAGCTCGCCAGAACCGTTATGATGTCGGCGCAAAAAACATTATCCAGAACGGGAGTGCGCCTTGAGCGACACGAATTATGCAGTGATTTACGACCTGCACAGCCATACCACAGCTTCCGATGGCTGCCTGACGCCAGAAGCATTGGTGCACCGTGCAGTCGATGATAAGCTGTCAAACATGAGAATTGTGCCTAATGAGTGAGCTAACTTACATTAATTGCGTTGCGCTCACTGCCCGCTTTCCAGTCGGGAAACCTGTCGTGCCAGCTGCATTAATGAATCGGCCAACGCGCGGGGAGAGGCGGTTTGCGTATTGGGCGCCAGGGTGGTTTTTCTTTTCACCAGTGAGACGGGCAACAGCTGATTGCCCTTCACCGCCTGGCCCTGAGAGAGTTGCAGCAAGCGGTCCACGCTGGTTTGCCCCAGCAGGCGAAAATCCTGTTTGATGGTGGTTAACGGCGGGATATAACATGAGCTGTCTTCGGTATCGTCGTATCCCACTACCGAGATATCCGCACCAACGCGCAGCCCGGACTCGGTAATGGCGCGCATTGCGCCCAGCGCCATCTGATCGTTGGCAACCAGCATCGCAGTGGGAACGATGCCCTCATTCAGCATTTGCATGGTTTGTTGAAAACCGGACATGGCACTCCAGTCGCCTTCCCGTTCCGCTATCGGCTGAATTTGATTGCGAGTGAGATATTTATGCCAGCCAGCCAGACGCAGACGCGCCGAGACAGAACTTAATGGGCCCGCTAACAGCGCGATTTGCTGGTGACCCAATGCGACCAGATGCTCCACGCCCAGTCGCGTACCGTCTTCATGGGAGAAAATAATACTGTTGATGGGTGTCTGGTCAGAGACATCAAGAAATAACGCCGGAACATTAGTGCAGGCAGCTTCCACAGCAATGGCATCCTGGTCATCCAGCGGATAGTTAATGATCAGCCCACTGACGCGTTGCGCGAGAAGATTGTGCACCGCCGCTTTACAGGCTTCGACGCCGCTTCGTTCTACCATCGACACCACCACGCTGGCACCCAGTTGATCGGCGCGAGATTTAATCGCCGCGACAATTTGCGACGGCGCGTGCAGGGCCAGACTGGAGGTGGCAACGCCAATCAGCAACGACTGTTTGCCCGCCAGTTGTTGTGCCACGCGGTTGGGAATGTAATTCAGCTCCGCCATCGCCGCTTCCACTTTTTCCCGCGTTTTCGCAGAAACGTGGCTGGCCTGGTTCACCACGCGGGAAACGGTCTGATAAGAGACACCGGCATACTCTGCGACATCGTATAACGTTACTGGTTTCACATTCACCACCCTGAATTGACTCTCTTCCGGGCGCTATCATGCCATACCGCGAAAGGTTTTGCACCATTCGATGGTGTCGAATTCGCGGCCGCTTCTAGAGCCGATGGCGCGCCGAGAGGCTTTACACTTTATGCTTCCGGCTCGTATAATGTGTGGAATTGTGAGCGGATAACAATTCCTAGGAATAATTTTGTTTAACTTTAAGAAGGAGATATACAT*ATG*TAAGCTAGCAGCTCGAGCAAATAAAACGAAAGGCTCAGTCGAAAGACTGGGCCTTTCGTTTTATCTGTTGTTTGTCGGTGAACGCTCTCCTGAGTAGGACAAATTACTAGTAGCGGCCGCTGCAG

*>pCl*

GAATTCGCGGCCGCTTCTAGAGTAACACCGTGCGTGTTGACTATTTTACCTCTGGCGGTGATAATGGTTGCCCTAGGAATAATTTTGTTTAACTTTAAGAAGGAGATATACAT*ATG*TAAGCTAGCAGCTCGAGCAAATAAAACGAAAGGCTCAGTCGAAAGACTGGGCCTTTCGTTTTATCTGTTGTTTGTCGGTGAACGCTCTCCTGAGTAGGACAAATTACTAGTAGCGGCCGCTGCAG

*>pJ23119*

GAATTCGCGGCCGCTTCTAGAGTTGACAGCTAGCTCAGTCCTAGGTATAATGCTAGCCCTAGGAATAATTTTGTTTAACTTTAAGAAGGAGATATACAT*ATG*TAAGCTAGCAGCTCGAGCAAATAAAACGAAAGGCTCAGTCGAAAGACTGGGCCTTTCGTTTTATCTGTTGTTTGTCGGTGAACGCTCTCCTGAGTAGGACAAATTACTAGTAGCGGCCGCTGCAG

*>RK2-oriT*

GGTACCTTTTCCGCTGCATAACCCTGCTTCGGGGTCATTATAGCGATTTTTTCGGTATATCCATCCTTTTTCGCACGATATACAGGATTTTGCCAAAGGGTTCGTGTAGACTTTCCTTGGTGTATCCAACGGCGTCAGCCGGGCAGGATAGGTGAAGTAGGCCCACCCGCGAGCGGGTGTTCCTTCTTCACTGTCCCTTATTCGCACCTGGCGGTGCTCAACGGGAATCCTGCTCTGCGAGGCTGGCCGGCTACCGCCGGCGTAACAGATGAGGGCAAGCGGATGGCTGATGAAACCAAGCCAACCAGGAAGGGCAGCCCACCTATCAAGGTGTACTGCCTTCCAGACGAACGAAGAGCGATTGAGGAAAAGGCGGCGGCGGCCGGCATGAGCCTGTCGGCCTACCTGCTGGCCGTCGGCCAGGGCTACAAAATCACGGGCGTCGTGGACTATGAGCACGTCCGCGAGCTGGCCCGCATCAATGGCGACCTGGGCCGCCTGGGCGGCCTGCTGAAACTCTGGCTCACCGACGACCCGCGCACGGCGCGGTTCGGTGATGCCACGATCCTCGCCCTGCTGGCGAAGATCGAAGAGAAGCAGGACGAGCTTGGCAAGGTCATGATGGGCGTGGTCCGCCCGAGGGCAGAGCCATGACTTTTTTAGCCGCTAAAACGGCCGGGGGGTGCGCGTGATTGCCAAGCACGTCCCCATGCGCTCCATCAAGAAGAGCGACTTCGCGGAGCTGGTGAAGTACATCACCGACGAGCAAGGCAAGACCGAAGCTT

*>CmR*

AAGCTTGCTTGGATTCTCACCAATAAAAAACGCCCGGCGGCAACCGAGCGTTCTGAACAAATCCAGATGGAGTTCTGAGGTCATTACTGGATCTATCAACAGGAGTCCAATAACTGCCTTAAAAAAATTACGCCCCGCCCTGCCACTCATCGCAGTACTGTTGTAATTCATTAAGCATTCTGCCGACATGGAAGCCATCACAGACGGCATGATGAACCTGAATCGCCAGCGGCATCAGCACCTTGTCGCCTTGCGTATAATATTTGCCCATAGTGAAAACGGGGGCGAAGAAGTTGTCCATATTGGCCACGTTTAAATCAAAACTGGTGAAACTCACCCAGGGATTGGCTGAGACGAAAAACATATTCTCAATAAACCCTTTAGGGAAATAGGCCAGGTTTTCACCGTAACACGCCACATCTTGCGAATATATGTGTAGAAACTGCCGGAAATCGTCGTGGTATTCACTCCAGAGCGATGAAAACGTTTCAGTTTGCTCATGGAAAACGGTGTAACAAGGGTGAACACTATCCCATATCACCAGCTCACCGTCTTTCATTGCCATACGGAACTCCGGATGAGCATTCATCAGGCGGGCAAGAATGTGAATAAAGGCCGGATAAAACTTGTGCTTATTTTTCTTTACGGTCTTTAAAAAGGCCGTAATATCCAGCTGAACGGTCTGGTTATAGGTACATTGAGCAACTGACTGAAATGCCTCAAAATGTTCTTTACGATGCCATTGGGATATATCAACGGTGGTATATCCAGTGATTTTTTTCTCCATTTTAGCTTCCTTAGCTCCTGAAAATCTCGATAACTCAAAAAATACGCCCGGTAGTGATCTTATTTCATTATGGTGAAAGTTGGAACCTCTTACGTGCCGATCAACGTCTCATTTTCGCCAGAGCTCGCCTGAATTC

*>p15A*

CTGCAGGGATATATTCCGCTTCCTCGCTCACTGACTCGCTACGCTCGGTCGTTCGACTGCGGCGAGCGGAAATGGCTTACGAACGGGGCGGAGATTTCCTGGAAGATGCCAGGAAGATACTTAACAGGGAAGTGAGAGGGCCGCGGCAAAGCCGTTTTTCCATAGGCTCCGCCCCCCTGACAAGCATCACGAAATCTGACGCTCAAATCAGTGGTGGCGAAACCCGACAGGACTATAAAGATACCAGGCGTTTCCCCCTGGCGGCTCCCTCGTGCGCTCTCCTGTTCCTGCCTTTCGGTTTACCGGTGTCATTCCGCTGTTATGGCCGCGTTTGTCTCATTCCACGCCTGACACTCAGTTCCGGGTAGGCAGTTCGCTCCAAGCTGGACTGTATGCACGAACCCCCCGTTCAGTCCGACCGCTGCGCCTTATCCGGTAACTATCGTCTTGAGTCCAACCCGGAAAGACATGCAAAAGCACCACTGGCAGCAGCCACTGGTAATTGATTTAGAGGAGTTAGTCTTGAAGTCATGCGCCGGTTAAGGCTAAACTGAAAGGACAAGTTTTGGTGACTGCGCTCCTCCAAGCCAGTTACCTCGGTTCAAAGAGTTGGTAGCTCAGAGAACCTTCGAAAAACCGCCCTGCAAGGCGGTTTTTTCGTTTTCAGAGCAAGAGATTACGCGCAGACCAAAACGATCTCAAGAAGATCATCTTATTAATCAGATAAAATATTACTAGATTTCAGTGCAATTTATCTCTTCAAATGTAGCACCTGAAGTCAGCCCCATACGATATAAGTTGTGGTACC

*>pSC101*

CTGCAGACGGGTTTTGCTGCCCGCAAACGGGCTGTTCTGGTGTTGCTAGTTTGTTATCAGAATCGCAGATCCGGCTTCAGGTTTGCCGGCTGAAAGCGCTATTTCTTCCAGAATTGCCATGATTTTTTCCCCACGGGAGGCGTCACTGGCTCCCGTGTTGTCGGCAGCTTTGATTCGATAAGCAGCATCGCCTGTTTCAGGCTGTCTATGTGTGACTGTTGAGCTGTAACAAGTTGTCTCAGGTGTTCAATTTCATGTTCTAGTTGCTTTGTTTTACTGGTTTCACCTGTTCTATTAGGTGTTACATGCTGTTCATCTGTTACATTGTCGATCTGTTCATGGTGAACAGCTTTAAATGCACCAAAAACTCGTAAAAGCTCTGATGTATCTATCTTTTTTACACCGTTTTCATCTGTGCATGTGGACAGTTTTCCCTTTGATATCTAACGGTGAACAGTTGTTCTACTTTTGTTTGTTAGTCTTGATGCTTCACTGATAGATACAAGAGCCATAAGAACCTCAGATCCTTCCGTATTTAGCCAGTATGTTCTCTAGTGTGGTTCGTTGTTTTTGCGTGAGCCATGAGAACGAACCATTGAGATCATGCTTACTTTGCATGTCACTCAAAAATTTTGCCTCAAAACTGGTGAGCTGAATTTTTGCAGTTAAAGCATCGTGTAGTGTTTTTCTTAGTCCGTTACGTAGGTAGGAATCTGATGTAATGGTTGTTGGTATTTTGTCACCATTCATTTTTATCTGGTTGTTCTCAAGTTCGGTTACGAGATCCATTTGTCTATCTAGTTCAACTTGGAAAATCAACGTATCAGTCGGGCGGCCTCGCTTATCAACCACCAATTTCATATTGCTGTAAGTGTTTAAATCTTTACTTATTGGTTTCAAAACCCATTGGTTAAGCCTTTTAAACTCATGGTAGTTATTTTCAAGCATTAACATGAACTTAAATTCATCAAGGCTAATCTCTATATTTGCCTTGTGAGTTTTCTTTTGTGTTAGTTCTTTTAATAACCACTCATAAATCCTCATAGAGTATTTGTTTTCAAAAGACTTAACATGTTCCAGATTATATTTTATGAATTTTTTTAACTGGAAAAGATAAGGCAATATCTCTTCACTAAAAACTAATTCTAATTTTTCGCTTGAGAACTTGGCATAGTTTGTCCACTGGAAAATCTCAAAGCCTTTAACCAAAGGATTCCTGATTTCCACAGTTCTCGTCATCAGCTCTCTGGTTGCTTTAGCTAATACACCATAAGCATTTTCCCTACTGATGTTCATCATCTGAGCGTATTGGTTATAAGTGAACGATACCGTCCGTTCTTTCCTTGTAGGGTTTTCAATCGTGGGGTTGAGTAGTGCCACACAGCATAAAATTAGCTTGGTTTCATGCTCCGTTAAGTCATAGCGACTAATCGCTAGTTCATTTGCTTTGAAAACAACTAATTCAGACATACATCTCAATTGGTCTAGGTGATTTTAATCACTATACCAATTGAGATGGGCTAGTCAATGATAATTACATGTCCTTTTCCCGGGAGATCTGGGTATCTGTAAATTCTGCTAGACCTTTGCTGGAAAACTTGTAAATTCTGCTAGACCCTCTGTAAATTCCGCTAGACCTTTGTGTGTTTTTTTTGTTTATATTCAAGTGGTTATAATTTATAGAATAAAGAAAGAATAAAAAAAGATAAAAAGAATAGATCCCAGCCCTGTGTATAACTCACTACTTTAGTCAGTTCCGCAGTATTACAAAAGGATGTCGCAAACGCTGTTTGCTCCTCTACAAAACAGACCTTAAAACCCTAAAGGCTTAAGTAGCACCCTCGCAAGCTCGGGCAAATCGCTGAATATTCCTTTTGTCTCCGACCATCAGGCACCTGAGTCGCTGTCTTTTTCGTGACATTCAGTTCGCTGCGCTCACGGCTCTGGCAGTGAATGGGGGTAAATGGCACTACAGGCGCCTTTTATGGATTCATGCAAGGAAACTACCCATAATACAAGAAAAGCCCGTCACGGGCTTCTCAGGGCGTTTTATGGCGGGTCTGCTATGTGGTGCTATCTGACTTTTTGCTGTTCAGCAGTTCCTGCCCTCTGATTTTCCAGTCTGACCACTTCGGATTATCCCGTGACAGGTCATTCAGACTGGCTAATGCACCCAGTAAGGCAGCGGTATCATCAACAGGCTTAGGTACC

*>repB*

GGTACCGAGCCTTTAGGCGAACTAGGGCGAAGCCCTTGGTCGCGGCGCAGCCGCCAGATGCTCAAATTTACTTGTTTATCCACAGGCGAAACCCTTTTTAATAGATCTTTCTTGTTTTTTAAGATCTTTCTTGTTTTTAGCGCCGCTGTAGCCCTTGGTGCTAAAGGGCTACAGCGGTTTAATGTGGAGAGATTTACGGTAAAACAGCCCCTAAAGGTGGAGAGATTTACGGTAAAACAATCCCTAAAGGTGGAGAGATTTACGGTGATATTCACAGTTAATTGTGGAGAACGTTGATAGCAATTCCACAATTGGTTATTGTGGAATCCCCAAAAATAAACTCCACAATAGTGACGTAAAATGAACAACCTATCTGTAACTAAATCAAATAGCTTGATAGATGCTAGTTACAAACTAAATGTACAAGCTCAAAAACTGGTCTTAGCTTGCCTCGGGAAAGTAGATTCGAGAGGTAATGCACCAAAGGAAATGACCCTAACAGCCCTTGAATTTTCAGAACTTATGGGGATAGACATCAAAAATGCTCATAGAGAGCTATACAAGGCGTCTGATACCCTTTTCGATGCCGTAATACTCCTAAGAGATGAGCAAGAAGAGGTAAAGCTGAGGTGGATTCAAAAAGGAGTGAAAAAACTCAAAGGACAAGGAGCAATAACAATCACTTGGACTGATGAAGTTTTGAAGTACATCAGTAGTTTACAGAGCAGGTTTACAACTTATAAGCTGCGACATATTGCGAATCTACAGTCAGCTCATTCTATTCGTTTATACGAACTTTTGATGAAATTTAACGCTACTGGAGAAAGAGTTATCTATGTGGATGACTTCCGCTCAGCATTAGGTATCTCCGACAAATACCCACAATTTAGAGACTTAAATAAATGGGTTATCAAGCCGGCCGTAGATGAACTAAATCAGCGTTCAGACCTCACCATAAATTACGAGACCATTAAGAAAGGGCGAACAGTGGCTGCATTGTCATTTGAGTTCAAGCAGAGCGCACAGCTAAAATTAGACGTATAATCAAATGCAACACACGCAACACCGCATCTCTTGATGCTGCAG

*>GFP*

CAT*ATG*CGTAAAGGAGAAGAACTTTTCACTGGAGTTGTCCCAATTCTTGTTGAATTAGATGGTGATGTTAATGGGCACAAATTTTCTGTCAGTGGAGAGGGTGAAGGTGATGCAACATACGGAAAACTTACCCTTAAATTTATTTGCACTACTGGAAAACTACCTGTTCCGTGGCCAACACTTGTCACTACTTTCGGTTATGGTGTTCAATGCTTTGCGAGATACCCAGATCACATGAAACAGCATGACTTTTTCAAGAGTGCCATGCCCGAAGGTTATGTACAGGAAAGAACTATATTTTTCAAAGATGACGGGAACTACAAGACACGTGCTGAAGTCAAGTTTGAAGGTGATACCCTTGTTAATAGAATCGAGTTAAAAGGTATTGATTTTAAAGAAGATGGAAACATTCTTGGACACAAATTGGAATACAACTATAACTCACACAATGTATACATCATGGCAGACAAACAAAAGAATGGAATCAAAGTTAACTTCAAAATTAGACACAACATTGAAGATGGAAGCGTTCAACTAGCAGACCATTATCAACAAAATACTCCAATTGGCGATGGCCCTGTCCTTTTACCAGACAACCATTACCTGTCCACACAATCTGCCCTTTCGAAAGATCCCAACGAAAAGAGAGACCACATGGTCCTTCTTGAGTTTGTAACAGCTGCTGGGATTACACATGGCATGGATGAACTATACAAA*TAA*GCTAGCGGCTCGAG

*>BFP*

CAT*ATG*TCTGAATTAATCAAAGAAAACATGCACATGAAATTATACATGGAAGGTACTGTTGATAACCACCACTTCAAATGTACTTCTGAAGGTGAAGGTAAACCATACGAAGGTACTCAAACTATGCGTATCAAAGTTGTTGAAGGTGGTCCATTACCATTCGCTTTCGATATCTTAGCTACTTCTTTCTTATACGGTTCTAAAACTTTCATCAACCACACTCAAGGTATCCCAGATTTCTTCAAACAATCTTTCCCAGAGGGCTTCACTTGGGAACGTGTTACTACTTACGAAGATGGTGGTGTTTTAACTGCTACTCAAGATACTTCTTTACAGGACGGTTGCCTGATCTACAACGTTAAAATCCGTGGTGTTAACTTCACTTCTAACGGTCCAGTTATGCAAAAAAAAACTTTAGGTTGGGAAGCGTTCACTGAAACTTTATACCCAGCTGACGGCGGTCTCGAAGGTCGTAATGATATGGCTTTAAAATTAGTTGGTGGTTCTCACTTAATCGCTAACATCAAAACTACTTACCGTTCTAAAAAACCAGCTAAAAACTTAAAAATGCCAGGTGTTTACTACGTTGATTACCGTTTAGAACGTATCAAAGAAGCTAACAACGAAACTTACGTTGAACAACACGAAGTTGCTGTTGCTCGTTACTGTGATTTACCATCTAAATTAGGTCACAAATTAAAC*TAATAA*GCTAGCGGCTCGAG

*>CFP*

CAT*ATG*CGTAAAGGTGAAGAATTATTCACTGGTGTTGTTCCAATCTTAGTTGAATTAGATGGTGATGTTAACGGTCACAAATTCTCTGTTCGTGGTGAAGGTGAAGGTGATGCTACTAACGGTAAATTAACTTTAAAATTCATCTGTACTACTGGTAAATTACCAGTTCCATGGCCAACTTTAGTTACTACTTTAACTTGGGGTGTTCAATGTTTCGCTCGTTACCCAGATCACATGAAACAACACGATTTCTTCAAATCTGCTATGCCAGAAGGTTACGTTCAAGAACGTACTATCTCTTTCAAAGATGATGGTACTTACAAAACTCGTGCTGAAGTTAAATTCGAAGGTGATACTTTAGTTAACCGTATCGAATTAAAAGGTATCGATTTCAAAGAAGATGGTAACATCTTAGGTCACAAATTAGAATACAACTTCAACTCTCACAACGTTTACATCACTGCTGATAAACAAAAAAACGGTATCAAAGCTAACTTCAAAATCCGTCACAACGTTGAAGATGGTTCTGTTCAATTAGCTGATCACTACCAACAAAACACTCCAATCGGTGATGGTCCAGTTTTATTACCAGATAACCACTACTTATCTACTCAATCTGTTTTATCTAAAGATCCAAACGAAAAACGTGATCACATGGTTTTATTAGAATTTGTTACTGCTGCTGGTATCACTCACGGTATGGATGAATTATACAAA*TAATAA*GCTAGCGGCTCGAG
